# Interspecies synergistic interactions mediated by cofactor exchange enhance stress tolerance by inducing biofilm formation

**DOI:** 10.1128/msystems.00884-24

**Published:** 2024-08-27

**Authors:** Lvjing Wang, Xiaoyu Wang, Hao Wu, Haixia Wang, Zhenmei Lu

**Affiliations:** 1 MOE Laboratory of Biosystem Homeostasis and Protection, College of Life Sciences, Zhejiang University, Hangzhou, China; 2 Cancer Center, Zhejiang University, Hangzhou, China; 3 College of Biotechnology and Bioengineering, Zhejiang University of Technology, Hangzhou, China; University of Minnesota Twin Cities, St. Paul, Minnesota, USA

**Keywords:** microbial interactions, hyperosmotic stress, biofilm formation, genome-scale metabolic models (GEMs), multi-omics analysis, DEHP

## Abstract

Metabolic exchange plays a crucial role in shaping microbial community interactions and functions, including the exchange of small molecules such as cofactors. Cofactors are fundamental to enzyme catalytic activities; however, the role of cofactors in microbial stress tolerance is unclear. Here, we constructed a synergistic consortium containing two strains that could efficiently mineralize di-(2-ethylhexyl) phthalate under hyperosmotic stress. Integration of transcriptomic analysis, metabolic profiling, and a genome-scale metabolic model (GEM) facilitated the discovery of the potential mechanism of microbial interactions. Multi-omics analysis revealed that the vitamin B_12_-dependent methionine-folate cycle could be a key pathway for enhancing the hyperosmotic stress tolerance of synergistic consortium. Further GEM simulations revealed interspecies exchange of S-adenosyl-L-methionine and riboflavin, cofactors needed for vitamin B_12_ biosynthesis, which was confirmed by *in vitro* experiments. Overall, we proposed a new mechanism of bacterial hyperosmotic stress tolerance: bacteria might promote the production of vitamin B_12_ to enhance biofilm formation, and the species collaborate with each other by exchanging cofactors to improve consortium hyperosmotic stress tolerance. These findings offer new insights into the role of cofactors in microbial interactions and stress tolerance and are potentially exploitable for environmental remediation.

**IMPORTANCE:**

Metabolic interactions (also known as cross-feeding) are thought to be ubiquitous in microbial communities. Cross-feeding is the basis for many positive interactions (e.g., mutualism) and is a primary driver of microbial community assembly. In this study, a combination of multi-omics analysis and metabolic modeling simulation was used to reveal the metabolic interactions of a synthetic consortium under hyperosmotic stress. Interspecies cofactor exchange was found to promote biofilm formation under hyperosmotic stress. This provides a new perspective for understanding the role of metabolic interactions in microbial communities to enhance environmental adaptation, which is significant for improving the efficiency of production activities and environmental bioremediation.

## INTRODUCTION

Microbes usually exist as members of communities in which metabolic interactions, also known as cross-feeding, are thought to be prevalent (1, 2). Cross-feeding underlies many positive interactions, such as synergistic interactions, in microbial communities and constitutes the primary driver of microbial community assembly (3, 4). Positive interactions among microorganisms are significant for enhancing the efficiency of productive activities and environmental remediation. Recent studies have suggested that abiotic stresses might be an important driving force for positive interactions (5, 6), such as the exchange of metabolites among complementary bacteria to relieve the effect of environmental stress on the community (7). Almost all microbes are exposed to self-produced or natural stress, which may explain why cross-feeding was observed to be prevalent. Given that environmental stress can facilitate the emergence of positive interactions, how microbial communities collaborate to adapt to environmental stress is therefore a matter of interest.

Biofilms are considered an effective form of bacterial tolerance to environmental stress (8, 9) and can be formed by single- or multispecies communities (10). Biofilms enable the exchange of metabolites (11), signaling molecules (12), and other compounds in close proximity, which can dictate the microbial interactions between microorganisms in communities. Interspecies interactions may promote biofilm formation (13, 14), but the molecular mechanisms involved remain unclear. Hyperosmolarity is a major abiotic stress that adversely affects the growth of microorganisms. To counteract these detrimental effects, bacteria can transport ions (e.g., Na^+^, K^+^, H^+^) or compatible solutes to prevent dehydration (15). Although the mechanisms of the hyperosmotic stress response in individual cells and biofilms have been well studied, the role of cross-feeding in bacterial hyperosmotic stress tolerance remains unknown.

The genome-scale metabolic model (GEM) is an emergent ecological modeling approach that can simulate the metabolic phenotypes of bacteria and predict metabolic exchange in microbial communities (16, 17). Schäfer et al. investigated the carbon source preferences of 224 bacterial strains in plant leaves and simulated pairwise interactions using GEMs. This work emphasized the benefits of ecological predictions using GEMs and provided guidance for the design of synthetic microbial communities based on substrates (18). In addition, GEMs have promising applications for the design and construction of synthetic microbial communities for bioremediation (19). In recent years, several studies have employed GEMs to investigate microbial metabolic interactions (20, 21) and optimal microbial combinations (22) for pollutant degradation. GEMs can help predict the interspecies exchange of substances that promote pollutant degradation and thus provide effective strategies for bioremediation.

Phthalate esters (PAEs) are chemicals used as plasticizers in a variety of products, such as agricultural films, food packaging, and medical devices (23), and have been identified as high-priority environmental pollutants in many countries (24, 25). Microbial degradation is considered a costless and effective strategy for environmental remediation. In recent decades, many PAE-degrading strains have been isolated and reported (26). Due to the widespread use of plastic agricultural films, agricultural soils have become common sites of PAE contamination (27), which leads to large amounts of PAE-degrading strains being distributed in the surface layer of agricultural soils. However, these degrading bacteria are frequently exposed to osmotic stress conditions caused by seasonal irrigation or drought. Hyperosmotic pressure affects bacterial growth in saline or contaminated soils, preventing them from performing critical metabolic processes such as biological control of plant diseases and pollutant decomposition (28). Given the serious risk posed by PAEs to human health, it is critical to develop effective strategies to remove PAEs from the surrounding environment.

Due to the resource-limited nature of pollutant degradation processes, non-degrading bacteria can only utilize downstream metabolites secreted by degrading bacteria; thus, metabolic interactions seem to be more prevalent in such systems, which have attracted the attention of researchers. In this study, we constructed a novel binary synergistic consortium for investigating how microbial interactions promote bacterial stress tolerance. To identify the key metabolic interaction mechanisms in this synergistic consortium, we integrated the transcriptome, metabolome, and two-species GEM data to investigate the transcriptional response and metabolic exchange of the synergistic consortium under stress conditions.

Here, we address two pending questions: (i) How do interspecies interactions promote biofilm formation? (ii) How does metabolic cross-feeding play a role in bacterial stress tolerance? Overall, our findings indicated that (i) cofactors might be critical for biofilm formation and that (ii) interspecies exchange of cofactors might drive synergistic interactions in the bacterial consortium under environmental stress. These findings not only illustrate a new facet of the role of cofactors in microbial interactions and stress tolerance but also provide novel strategies for constructing synthetic microbial communities and enhancing microbial remediation under environmental stress.

## MATERIALS AND METHODS

### Strain isolation, chemicals, and culture conditions

After enrichment with gradually increasing concentrations of di-(2-ethylhexyl) phthalate (DEHP), one of the most common PAEs, a highly efficient DEHP-degrading strain, *Rhodococcus ruber* ZM15, was isolated from agricultural surface layer soil covered with plastic film (Hangzhou, Zhejiang, China). Dozens of bacteria that do not degrade DEHP were isolated from the same enrichment. After pairwise combinations with the degrading strain at an OD_600_ ratio of 1:1, a non-degrading strain, *Epilithonimonas zeae* ZM18, that could help the degrading strain tolerate multiple environmental stresses was screened. The strains were grown at 30°C with agitation at 200 rpm.

All PAEs used in this study were of analytical grade and purchased from Aladdin Biochemical Technology Co., Ltd. (Shanghai, China). Organic solvents, such as methanol and ethyl acetate, were of HPLC grade and obtained from Sinopharm Chemical Reagent Co., Ltd. (Shanghai, China). S-Adenosyl-L-methionine (SAM), riboflavin (RIBF), and 2,3-dihydroxybenzoic acid were purchased from Macklin Inc. (Shanghai, China).

Minimal salt medium (MSM) supplemented with 1,000 mg/L DEHP was used for enrichment and cultivation. The MSM (29) (per liter) contained 5.8 g K_2_HPO_4_, 4.5 g KH_2_PO_4_, 2.0 g (NH_4_)_2_SO_4_, 0.34 g MgCl_2_·6H_2_O, and 1 mL trace metal stock solution. The trace metal stock solution (per liter) contained 0.15 g MnCl_2_·4H_2_O, 0.18 g FeSO_4_·7H_2_O, 0.24 g Na_2_MoO_4_·2H_2_O, and 2.0 g CaCl_2_. Lysogeny broth (LB) medium, which contained 10 g of tryptone, 5 g of yeast extract, and 10 g of NaCl (per liter), was used to cultivate non-degrading bacteria and activate frozen strains. The solid medium was prepared by solidifying lipid medium with 1.7% agar. All media were adjusted to pH 7.0 and autoclaved at 121°C for 20 min before use.

### Analysis of DEHP degradation

The DEHP-degrading strain was inoculated in MSM containing 1,000 mg/L DEHP, and the residual DEHP was extracted using an equal volume of ethyl acetate and detected by HPLC (Agilent 2000, USA) (29). After 12, 24, 36, 48, 60, and 72 hours of incubation, the supernatants of the cultures were frozen at −80°C and then lyophilized by a freeze dryer. The powder was dissolved in 15 mL of methanol to concentrate the metabolites. The metabolites produced during the DEHP degradation process were identified by HPLC-MS (AB Sciex Triple TOF 5600+, USA) (30). The chromatographic column was a reversed-phase C18 column, and the mobile phases were methanol (A) and ultrapure water containing 0.1% formic acid (B). The gradient elution conditions were as follows: 0–10 min, 60% B; 11–35 min, 60%–100% B; 36–40 min, 100%–100% B; and 41–51 min, 100%–15% B. The flow rate was 1 mL/min, the injection volume was 10 µL, and the column temperature was 40°C. The mass spectrometry parameters were as follows: source voltage of 5.5 kV (positive) and 4.5 kV (negative); temperature of 600°C (positive) and 550°C (negative); air and nitrogen pressures of 50 and 35 psi, respectively; de-clustering potential of 100 V; collision energy of 10 V; and MS/MS scanning range of 50–1,000 m/z. The mass spectrometry data were analyzed and processed with PeakView v1.2. The structure of the metabolites was inferred by analyzing the profiles, and the metabolic pathway of DEHP was proposed.

### Drop plate experiments

The activated strains were prepared as seed solutions with an initial OD_600_ of 0.05, serially diluted. and dropped on plates under various stress conditions. Except for the oligotrophic condition, which was formulated as MSM with 0.2% glucose, all other stress conditions were based on LB media.

### Genome sequencing, assembly, and annotation

Genomic DNA was extracted using the sodium dodecyl sulfate method, and the harvested DNA was detected by agarose gel electrophoresis and quantified by a Qubit 2.0 Fluorometer (Thermo Fisher Scientific, USA). The whole genome was sequenced using the Nanopore PromethION platform and Illumina NovaSeq PE150 by Beijing Novogene Bioinformatics Technology Co., Ltd. (Beijing, China).

Nanopore and PE150 data were combined with Unicycler (31) for assembly, and the reads were then compared to the assembled sequence to determine the distribution of sequencing depth and whether the genome was chromosomal or circular. Genome component prediction included the prediction of coding genes, repetitive sequences, non-coding RNA, genomic islands, transposons, prophages, and clustered regularly interspaced short palindromic repeats. Gene function was predicted using seven databases, including GO (Gene Ontology), KEGG (Kyoto Encyclopedia of Genes and Genomes), COG (Clusters of Orthologous Groups), NR (Non-Redundant Protein Database), TCDB (Transporter Classification Database), and Swiss-Prot. The predicted genes were compared with each functional database by BLAST (blastp, *e*-value ≤1e−5), and the comparison with the highest score (default identity ≥40%, coverage ≥40%) was selected for annotation.

The raw sequencing data have been deposited in the NCBI database with the BioProject accession numbers PRJNA730359 and PRJNA730364. The genomes of *R. ruber* ZM15 and *E. zeae* ZM18 have been deposited in GenBank under accession numbers GCA_023278385.1 and GCA_023278365.1.

### Biofilm formation assay

To examine biofilm formation, two strains were mixed at 1:1 at an initial OD_600_ of 0.01 and inoculated in 2 mL of MSM supplemented with DEHP in 96-well microplates, 24-well microplates (Corning Costar, flat bottom, USA), or confocal dishes (Cellvis, 35 mm, USA). The monoculture system was inoculated with only one strain in the same way.

Biofilm formation was quantified by crystal violet staining. The biofilms in the confocal dishes were stained with a LIVE/DEAD *Bac*Light Bacterial Viability and Counting Kit (Thermo Fisher Scientific, USA) and observed under a confocal laser scanning microscope (CLSM) (Olympus FV3000, Japan). The SYTO 9 in the staining solution could bind to the nucleic acids of live cells and fluoresce green, with an excitation wavelength of 488 nm and an emission wavelength of 530 nm.

### RNA-seq analysis

Total RNA was obtained from monocultures or cocultures of MSM supplemented with 1,000 mg/L DEHP under hyperosmotic stress (0%, 2.5%, or 5% NaCl) using an RNeasy Mini Kit (QIAGEN, Germany). RNA sequencing was performed at Beijing Novogene Bioinformatics Technology Co., Ltd. (Beijing, China).

RNA degradation and contamination were monitored on 1% agarose gels, and RNA integrity was assessed using the RNA Nano 6000 Assay Kit of the Bioanalyzer 2100 system (Agilent, USA). Total RNA was used as input material for RNA sample preparation. To preferentially select cDNA fragments 370–420 bp in length, the library fragments were purified with the AMPure XP system (Beckman Coulter, Beverly, USA). Library quality was assessed on an Agilent Bioanalyzer 2100 system. The library preparations were sequenced on an Illumina NovaSeq platform, and 150 bp paired-end reads were generated. Raw data (raw reads) in fastq format were first processed through in-house Perl scripts. In this step, clean data (clean reads) were obtained by removing reads containing adapters, reads containing N bases, and low-quality reads from the raw data. At the same time, the *Q*20, *Q*30, and GC contents of the clean data were calculated. All downstream analyses were based on high-quality clean data. The reference genome and gene model annotation files were downloaded from the genome website. Both the construction of the index of the reference genome and the alignment of the clean reads to the reference genome were performed using Bowtie2-2.2.3.

Differentially expressed genes (DEGs) were identified using the DESeq2 R package with a threshold of |log_2_ Fold Change| > 1 and adjusted *P* < 0.05.

The raw sequencing data have been deposited in the NCBI database with BioProject accession number PRJNA1004036.

### qRT-PCR

Total RNA preparations were treated with gDNA Eraser (Takara, Japan) and reverse transcribed to cDNA using the PrimeScript RT Kit (Takara, Japan). The primers used were designed with Beacon Designer 7 software (Table S1). The transcript levels of the genes involved in vitamin B_12_ biosynthesis were measured by qRT-PCR using TransStart Top Green qPCR SuperMix (TransGen, China) on a Rotor-Gene Q Detection System (QIAGEN, Germany) with a three-step method. The 16S rRNA gene V3 region was used as an internal control, and the relative expression of these genes was determined by the comparative threshold cycle (average amplification efficiency)^−ΔCT^ method (32).

### Extracellular metabolome analysis

The supernatants of cells monocultured or cocultured in MSM supplemented with 1,000 mg/L DEHP under hyperosmotic stress (0%, 2.5%, 5% NaCl) were collected and analyzed by UHPLC-MS/MS by Beijing Novogene Bioinformatics Technology Co., Ltd. (Beijing, China). More than 5 mL of the culture sample was centrifuged at 3,000 rpm at 4°C for 10 min, and all the supernatant was frozen in liquid nitrogen for 15 min and then put it into a refrigerator at −80°C.

One milliliter of the supernatant sample was lyophilized in a lyophilizer, and 100 µL of 80% aqueous methanol was added. The sample was vortexed and shaken, placed in an ice bath for 5 min, and centrifuged at 15,000 g, 4°C for 15 min. The supernatant was diluted in 53% methanol (prepared in mass spectrometry-grade water). The sample was centrifuged at 15,000 g, 4°C for 15 min, and the supernatant was collected and injected into the sample for LC-MS analysis. UHPLC-MS/MS analyses were performed using a Vanquish UHPLC system (Thermo Fisher, Germany) coupled with an Orbitrap Q Exactive HF-X mass spectrometer (Thermo Fisher, Germany). The raw data files generated by UHPLC-MS/MS were processed using Compound Discoverer 3.1 (CD3.1, Thermo Fisher) to perform peak alignment, peak picking, and quantitation for each metabolite. Peaks were matched with the mzCloud (https://www.mzcloud.org/), mzVault, and MassList databases to obtain accurate qualitative and relative quantitative results. The metabolites were annotated using the KEGG database (https://www.genome.jp/kegg/pathway.html), HMDB (https://hmdb.ca/metabolites), and LIPID Maps database (http://www.lipidmaps.org/).

Univariate analysis (*t*-test) was applied to calculate the statistical significance (*P*-value). The metabolites with VIP > 1 and *P*-value < 0.05 and |log_2_ Fold Change| > 1 were considered differentially abundant metabolites. For clustering heatmaps, the data were normalized using *z*-scores of the intensity areas of differentially abundant metabolites and plotted by the Pheatmap package in R. Raw sequencing data were deposited in the MetaboLights database (https://www.ebi.ac.uk/metabolights/) with the identifier MTBLS8399.

### Genome-scale metabolic model reconstruction and interspecies metabolic exchange analysis

Pathway Tools 26.0 software (33) was used to reconstruct and refine the single-species GEMs for *R. ruber* ZM15 and *E. zeae* ZM18. OrthoFinder was used to determine protein homology with phylogenetically close strains (34). The accuracy and quality of GEMs were validated using Biolog PM1 Microplates (BIOLOG, USA). The biomass composition was determined according to the literature and experimental data, and the determination method was based on that described by Hu et al. (20). The available models in SBML format and MEMOTE (35) reports for *R. ruber* ZM15 and *E. zeae* ZM18 have been uploaded to GitHub (https://github.com/Wang-Lvjing/GEMs-for-R.-ruber-ZM15-and-E.-zeae-ZM18.git).

Based on two single-strain GEMs, a two-strain GEM was reconstructed, in which the metabolites were localized to the following five compartments: (i) [*r*] defined the cytoplasm of *R. ruber* ZM15; (ii) [*t*] defined the extracellular space of *R. ruber* ZM15; (iii) [*z*] defined the cytoplasm of *E. zeae* ZM18; (iv) [*s*] defined the extracellular space of *E. zeae* ZM18; and (v) [*e*] defined the extracellular space shared by the two strains. The metabolic exchange potential of the two-species consortium was analyzed using parsimonious flux balance analysis (pFBA) (36) with COBRA v3.0 (37). Details of the reconstruction and refinement processes for the GEMs are provided in the supplemental information (Fig. S1). The two-species GEM is provided in the Supplemental data set.

### Extracellular polymeric substances extraction and analysis

A volume of 35 mL of culture was measured and centrifuged at 4°C and 5,000 g for 15 min, after which the supernatant was removed. Bacteria were resuspended in 0.9% NaCl solution, held in a water bath at 60°C for 30 min, and then centrifuged at 4°C and 10,000 g for 15 min. The centrifuged supernatant was filtered through 0.45 µm mixed cellulose membranes and dialyzed in purified water for 24 hours using a 3.5 kD dialysis bag (Shanghai yuanye Bio-Technology Co., Ltd, China), and the extracellular polymer substance (EPS) solution was stored at −20°C (38). Proteins, polysaccharides, and DNA were determined by the Bradford, phenol-sulfuric acid, and diphenylamine methods, respectively.

### Microscopy

Strains were cultured in MSM supplemented with 1,000 mg/L DEHP, collected by centrifugation, and washed three times with phosphate-buffered saline (pH 7.4). The bacteria were fixed by double fixation with glutaraldehyde and osmium acid, dried by ethanol gradient dehydration and the critical point drying method, and finally gold sprayed and observed by scanning electron microscopy (Zeiss GeminiSEM 300, Germany).

Colonies on solid DEHP-MSM plates with 0% NaCl and 5% NaCl were dipped and resuspended in 20 µL ddH_2_O, and then 4 µL dilutions were transferred to a transmission electron microscopy (TEM) carrier and stained with uranium acetate. The extracellular matrix and cell-cell contacts were observed using TEM (JEOL JEM-1400flash, Japan).

### Statistical analysis

Analysis was carried out with R and GraphPad Prism 8.0.1. Figures were produced using GraphPad Prism 8.0.1, Hiplot (https://hiplot.cn/) (39), and Chiplot (https://www.chiplot.online/). ChemSketch was used to draw the structural formulas of compounds.

## RESULTS

### Construction of a binary synergistic consortium

**Fig 1:**
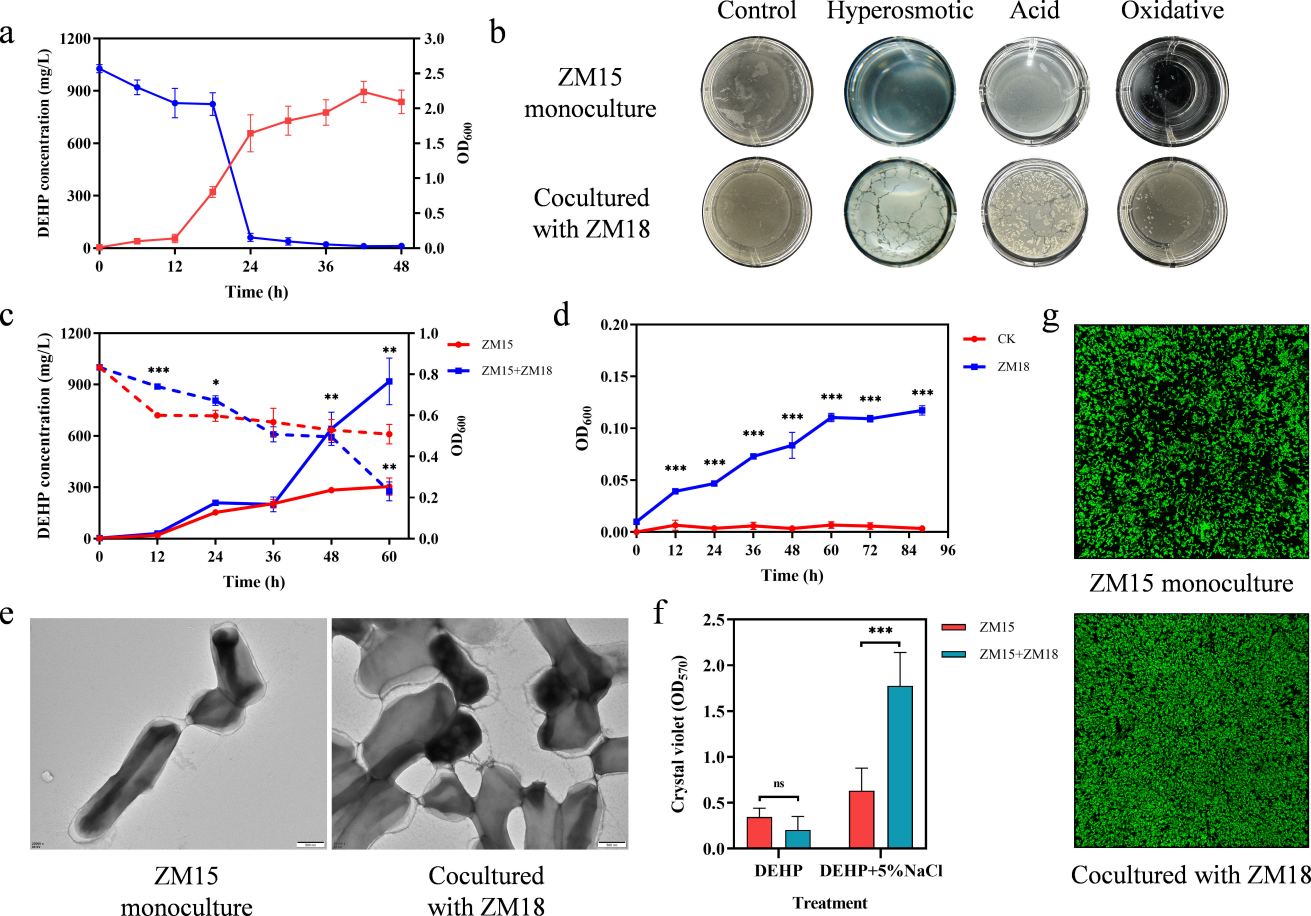
Characterization of the synergistic consortium during DEHP degradation under hyperosmotic stress. (a) The growth-degradation curve of *R. ruber* ZM15 on DEHP. (b) The growth of *R. ruber* ZM15 monoculture and coculture with *E. zeae* ZM18 under various conditions. (c) Comparison of the growth-degradation curves of *R. ruber* ZM15 monocultured or cocultured with *E. zeae* ZM18 under hyperosmotic stress (5% NaCl). The solid lines indicate the growth of the monoculture (red) or coculture (blue); the dotted lines indicate DEHP degradation in the monoculture (red) or coculture (blue). Significance tests were performed using a two-tailed *t*-test via GraphPad Prism 8.0.1. The symbol *** indicates *P* < 0.001, ** indicates *P* < 0.01, and * indicates *P* < 0.05. (d) The growth curve of *E. zeae* ZM18 when cultured in *R. ruber* ZM15 spent medium. The CK group represents spent medium that was not inoculated with *E. zeae* ZM18 to exclude media contamination. Significance tests were performed using a two-tailed *t*-test via GraphPad Prism 8.0.1. The symbol *** indicates *P* < 0.001. Error bars were calculated based on three biological replicates of each group (**a, c, and **d). (e) Extracellular morphology of *R. ruber* ZM15 monocultured or cocultured with *E. zeae* ZM18 under hyperosmotic stress observed by TEM. (f) The amount of biofilm formation in the *R. ruber* ZM15 monoculture and coculture with *E. zeae* ZM18 was determined by the crystal violet method. Significance tests were performed using a two-tailed *t*-test via GraphPad Prism 8.0.1. The symbol *** indicates *P* < 0.001, and ns indicates *P* ≥ 0.05. Error bars were calculated based on six biological replicates of each group. (g) Biofilm phenotypes of *R. ruber* ZM15 monoculture or coculture with *E. zeae* ZM18 under hyperosmotic stress were observed by CLSM.

The synergistic consortium was composed of two strains. First, the DEHP-degrading strain *R. ruber* ZM15 was isolated from soil covered with plastic film using a long-term domestication strategy (Fig. S2a). *R. ruber* ZM15 could utilize DEHP as the sole carbon source and could completely degrade 1,000 mg/L DEHP within 48 hours with an OD_600_ of 2 (Fig. 1a). It was also found that some PAEs with higher molecular weights, such as dibutyl phthalate, diisobutyl phthalate, benzyl butyl phthalate, and diisodecyl phthalate, could be efficiently degraded by strain ZM15 within 48 hours, while low-molecular-weight PAEs, such as dimethyl phthalate and diethyl phthalate, could not be utilized (data not shown). According to the whole genome and transcriptome of *R. ruber* ZM15, the genetic basis and possible pathway of DEHP degradation were identified (Fig. S2b through d). The metabolic pathways of DEHP in *R. ruber* ZM15 were proposed through metabolic intermediate identification using HPLC-MS (Fig. S3 and S4). Despite the excellent stress tolerance of *R. ruber* ZM15 in enriched medium (Fig. S5), the growth of *R. ruber* ZM15 was significantly inhibited by various stresses during DEHP degradation (Fig. 1b).

Second, *E. zeae* ZM18 was derived from pairwise selection from a library of interacting bacteria of the degrading consortium with the aim of screening for strain capable of relieving the stress of *R. ruber* ZM15. *E. zeae* ZM18 could not utilize DEHP as the sole carbon source but could significantly enhance the growth of *R. ruber* ZM15 under various stresses (5% NaCl, pH 5.5, and 50 mM H_2_O_2_) (Fig. 1b and c). We further explored their interaction mechanism under hyperosmotic stress (5% NaCl). The growth of *E. zeae* ZM18 was significantly enhanced by the spent medium from the incubation system of *R. ruber* ZM15 (Fig. 1d). These results indicated strong metabolic facilitation between the two strains. We noticed that the bacteria tended to flocculate during incubation and therefore speculated that many extracellular polymers might have been secreted and that these two strains might form denser biofilms to respond to environmental stress. According to previous studies (15), microorganisms can tolerate environmental stresses by living in single- or multiple-species biofilms. Further experiments verified that cocultures of *R. ruber* ZM15 and *E. zeae* ZM18 could indeed form denser biofilms synergistically under hyperosmotic stress (Fig. 1e through g). These results suggested that *E. zeae* ZM18 could stimulate *R. ruber* ZM15 to form biofilms, restore growth capacity, and enhance DEHP degradation under hyperosmotic stress.

### Vitamin B_12_ might be critical for hyperosmotic stress tolerance

**Fig 2:**
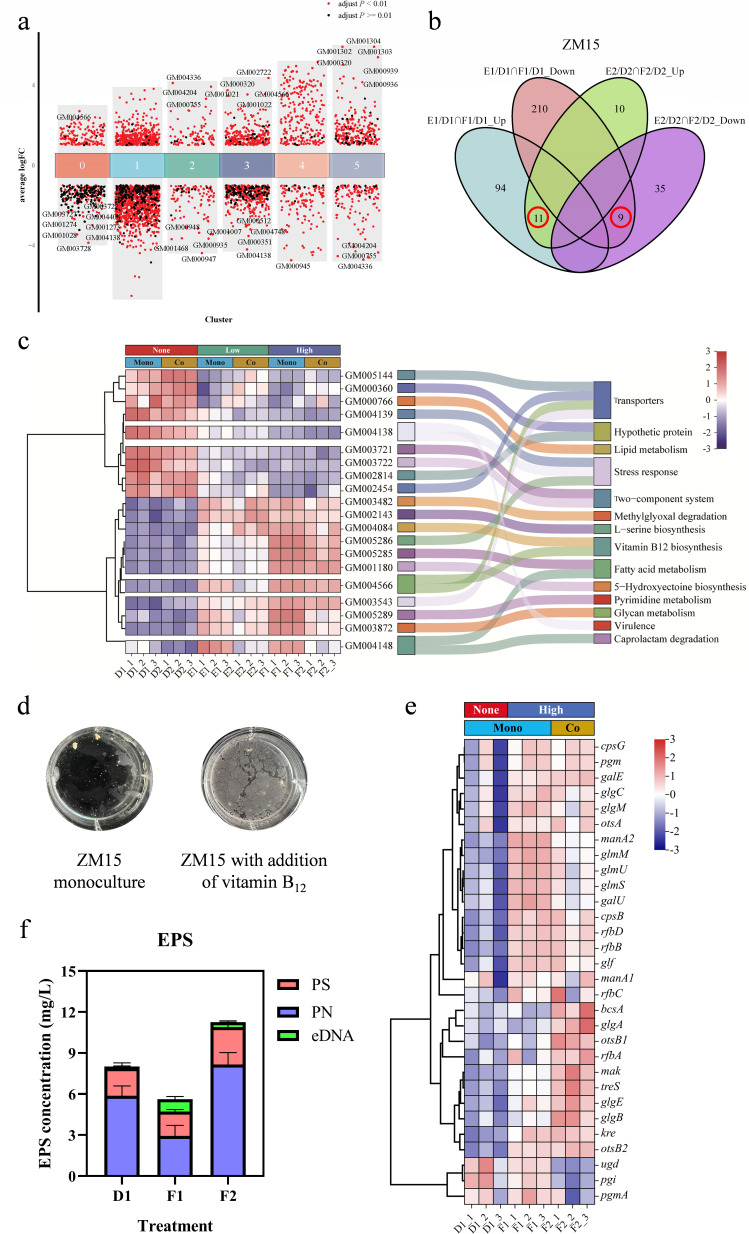
Transcriptional response of *R. ruber* ZM15 to hyperosmotic stress in the synergistic consortium. (a) Differential gene expression analysis showing up- and downregulated genes across all clusters. Adjusted *P*-values <0.01 are indicated in red, while adjusted *P*-values ≥ 0.01 are indicated in black. (b) Venn diagram depicting the overlap and distribution of co-up/downregulated DEGs of *R. ruber* ZM15 under both low- (2.5% vs 0%) and high- (5% vs 0%) salt conditions in monoculture and coculture, respectively. D1, *R. ruber* ZM15 monoculture under no-salt condition in DEHP-MSM; E1, *R. ruber* ZM15 monoculture under low-salt condition (2.5% NaCl) in DEHP-MSM; F1, *R. ruber* ZM15 monoculture under high-salt condition (5% NaCl) in DEHP-MSM; D2, *R. ruber* ZM15 coculture with *E. zeae* ZM18 under no-salt condition in DEHP-MSM; E2, *R. ruber* ZM15 coculture with *E. zeae* ZM18 under low-salt condition (2.5% NaCl) in DEHP-MSM; F2, *R. ruber* ZM15 coculture with *E. zeae* ZM18 under high-salt condition (5% NaCl) in DEHP-MSM. The same as in all the figures in this study. (c) Heatmap analysis of the 20 co-up/downregulated genes under both low- and high-salt conditions in both monoculture and coculture. The heatmaps showed the log_2_ (fragments per kilobase of exon per million mapped fragments) values of each DEG. The data were normalized using StandardScaler, and the Ward’s clustering method was used for heatmap analysis. The same as in Fig. 3 and 4. None indicates 0% NaCl salt condition, Low indicates 2.5% NaCl salt condition, and High indicates 5% NaCl salt condition. Mono indicates monoculture of *R. ruber* ZM15, and Co indicates *R. ruber* ZM15 and *E. zeae* ZM18 coculture. The same as in all the figures in this study. The Sankey diagram shows the KEGG pathways of these 20 DEGs in *R. ruber* ZM15. (d) The growth of *R. ruber* ZM15 monoculture and with the addition of a low concentration (0.01% of the DEHP molar concentration) of vitamin B_12_. (e) Heatmap analysis of the biofilm formation-related genes in *R. ruber* ZM15. (f) EPS production of different treatments. PS indicates extracellular polysaccharide, PN indicates extracellular protein, and eDNA indicates extracellular DNA. Error bars were calculated based on three biological replicates of each group.

To reveal the hyperosmotic stress response mechanism of the DEHP-degrading consortium, the transcriptomes of both monocultures and cocultures with and without salt addition were examined. A total of 420 DEGs or 1,615 DEGs of *R. ruber* ZM15 were significantly differentially expressed (|log_2_ Fold Change| > 1, adjusted *P* < 0.05) under low- (2.5% NaCl) or high- (5% NaCl) salt conditions, respectively, in monoculture (Fig. 2a; Fig. S6a). Many of these DEGs were downregulated, indicating that the growth and biochemical activities of *R. ruber* ZM15 were significantly inhibited under hyperosmotic stress (Fig. S6a). In addition, we found that 204 DEGs or 728 DEGs of *R. ruber* ZM15 were significantly differentially expressed (|log_2_ Fold Change| > 1, adjusted *P* < 0.05) under low- or high-salt conditions, respectively, during coculture (Fig. 2a; Fig. S6b). The number of DEGs under hyperosmotic stress was lower in coculture than in monoculture, indicating that *E. zeae* ZM18 helped to restore the physiological and biochemical activities of *R. ruber* ZM15 at the transcriptional level under hyperosmotic stress. A total of 122 DEGs of *E. zeae* ZM18 were upregulated under both low- and high-salt conditions in coculture (Fig. S7). Most of these DEGs were involved in secondary metabolites and amino acid synthesis (Fig. S7d). In addition, genes involved in the synthesis of compatible solutes (*lysC*, *glgABC*) were upregulated in *E. zeae* ZM18 under hyperosmotic stress. Overall, we showed that *E. zeae* ZM18 induces the biofilm formation of *R. ruber* ZM15 through three pathways.

First, we found that vitamin B_12_, which could induce biofilm formation in *R. ruber* ZM15, might be critical for hyperosmotic stress tolerance. In monoculture, most of the pathways were downregulated under hyperosmotic stress (Fig. S6c). The significantly upregulated pathways of cofactor biosynthesis and porphyrin metabolism suggested that vitamin synthesis might be affected by hyperosmotic stress (Fig. S6c). By performing weighted gene co-expression network analysis, we identified 16 co-expressed gene modules of *R. ruber* ZM15 and explored the association between the gene modules and the phenotype of interest (Fig. S8a). Among them, the green module had the highest correlation with salt concentration (Fig. S8b). Three of the top 11 genes with the highest correlation in this module belong to the vitamin B_12_ biosynthesis pathway (Table S2). Compared with no-stress condition, only 11/9 DEGs were up/downregulated under both low- and high-salt conditions (Fig. 2b). Heatmap analysis revealed that the abundance of these 11 co-upregulated genes increased with increasing salt concentration, and 2 of these 11 genes were involved in vitamin B_12_ biosynthesis (Fig. 2c; Table S3). Moreover, cobalt ion uptake and efflux genes showed opposite trends with increasing salt concentration (Table S3).

Intriguingly, most of the genes involved in the vitamin B_12_ biosynthesis pathway were upregulated under hyperosmotic stress during monoculture but downregulated in coculture under hyperosmotic stress (Fig. S9; Table S4). qRT-PCR assays confirmed that most of these genes involved in the vitamin B_12_ biosynthesis pathway were upregulated under hyperosmotic stress but downregulated in coculture, consistent with the transcriptome data (Fig. S10). The reduced vitamin B_12_ synthesis in *R. ruber* ZM15 in coculture may be due to interspecies cofactor exchange. Through exogenous addition experiments, we found that vitamin B_12_ could promote the biofilm formation in *R. ruber* ZM15 (Fig. 2d). These results implied that vitamin B_12_ might profoundly influence the hyperosmotic stress tolerance of *R. ruber* ZM15.

**Fig 3:**
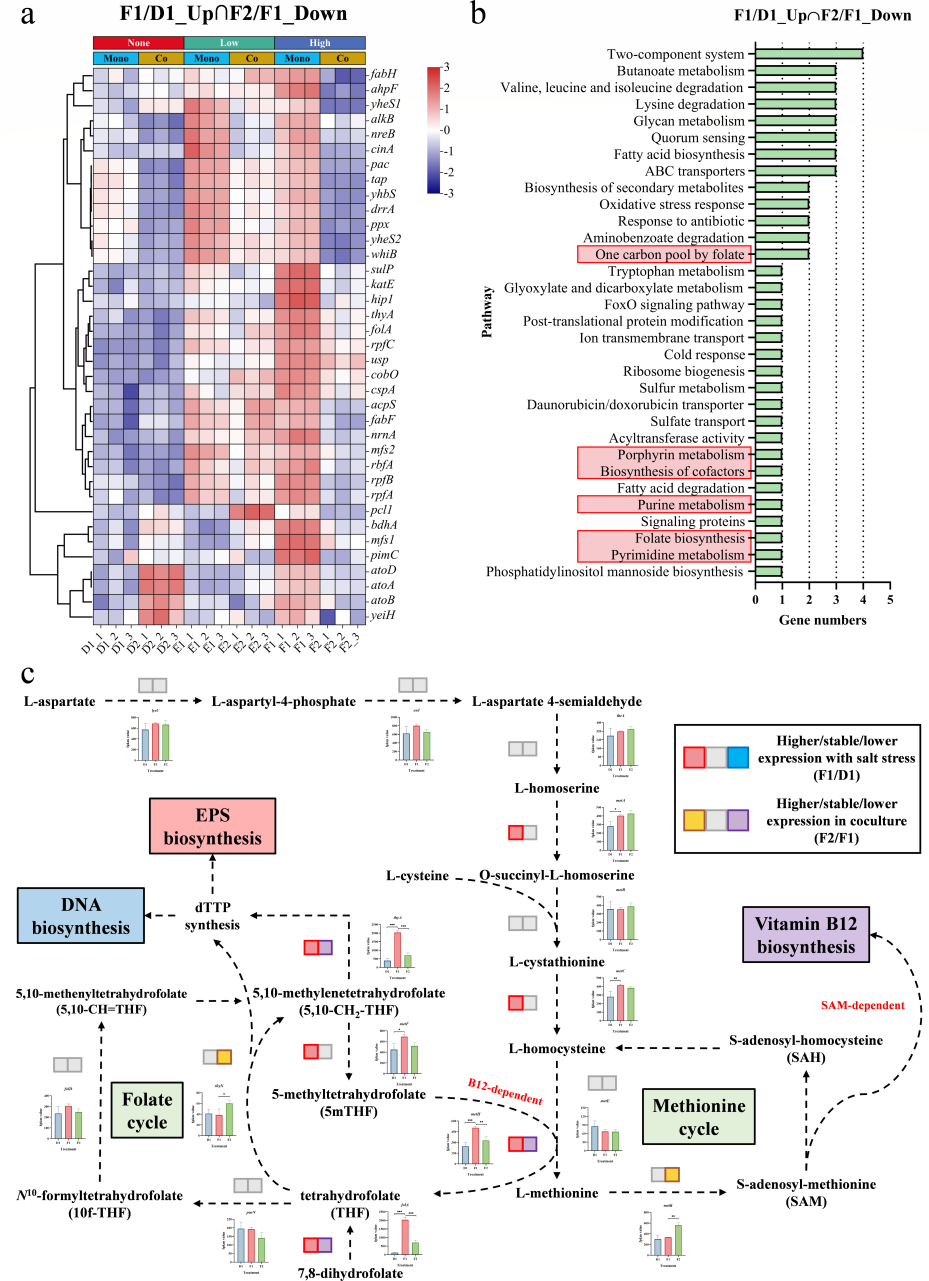
Transcriptional analysis of DEGs that showed opposite trends under hyperosmotic stress and in coculture. (a) Heatmap analysis of the genes upregulated under hyperosmotic stress (F1 vs D1) but downregulated in coculture (F2 vs F1). (b) KEGG pathway analysis of these DEGs that showed opposite trends under hyperosmotic stress and in coculture. (c) Changes in the transcriptomic data of genes involved in the methionine-folate cycle in *R. ruber* ZM15. Significance tests were performed using a two-tailed *t*-test via GraphPad Prism 8.0.1. The symbol *** indicates *P* < 0.001, ** indicates *P* < 0.01, and * indicates *P* < 0.05. Each group had three biological replicates.

Second, we found that the presence of *E. zeae* ZM18 upregulated the expression of biofilm matrix synthesis genes compared to the monoculture condition of *R. ruber* ZM15. Exopolysaccharide synthesis includes the synthesis of glycogen, cellulose, and enterobacterial common antigen (ECA). In *R. ruber* ZM15, hyperosmotic stress resulted in upregulated expression of biofilm matrix synthesis genes, and coculture promoted further upregulation of the expression of these genes (Fig. 2e). We extracted extracellular polymer substance, and the results showed that EPS production was greater in coculture under hyperosmotic stress (Fig. 2f). Furthermore, we analyzed the DEGs that showed opposite trends under hyperosmotic stress and in coculture conditions. There were 49/71 DEGs that were up/downregulated under hyperosmotic stress (F1 vs D1) but down/upregulated in coculture (F2 vs F1) (Fig. 3a). Among these 49 DEGs, genes involved in folate biosynthesis, pyrimidine metabolism, purine metabolism, and porphyrin metabolism (Fig. 3b) are involved in the vitamin B_12_ biosynthesis pathway as well as the methionine-folate cycle and ultimately affect DNA biosynthesis. Extracellular DNA (eDNA) is an important component of EPS, and vitamin B_12_ may affect eDNA synthesis by regulating the methionine-folate cycle. dTTP not only served as a precursor for DNA biosynthesis but also participated in the biosynthesis of dTDP-β-L-rhamnose (*pgm*, *rfbABCD*, *kre*) (Fig. 3b), as a glycan component of the ECA in biofilm formation. This suggested that vitamin B_12_ may enhance DNA and EPS biosynthesis in response to hyperosmotic stress via methionine-folate cycle (Fig. 3c).

**Fig 4:**
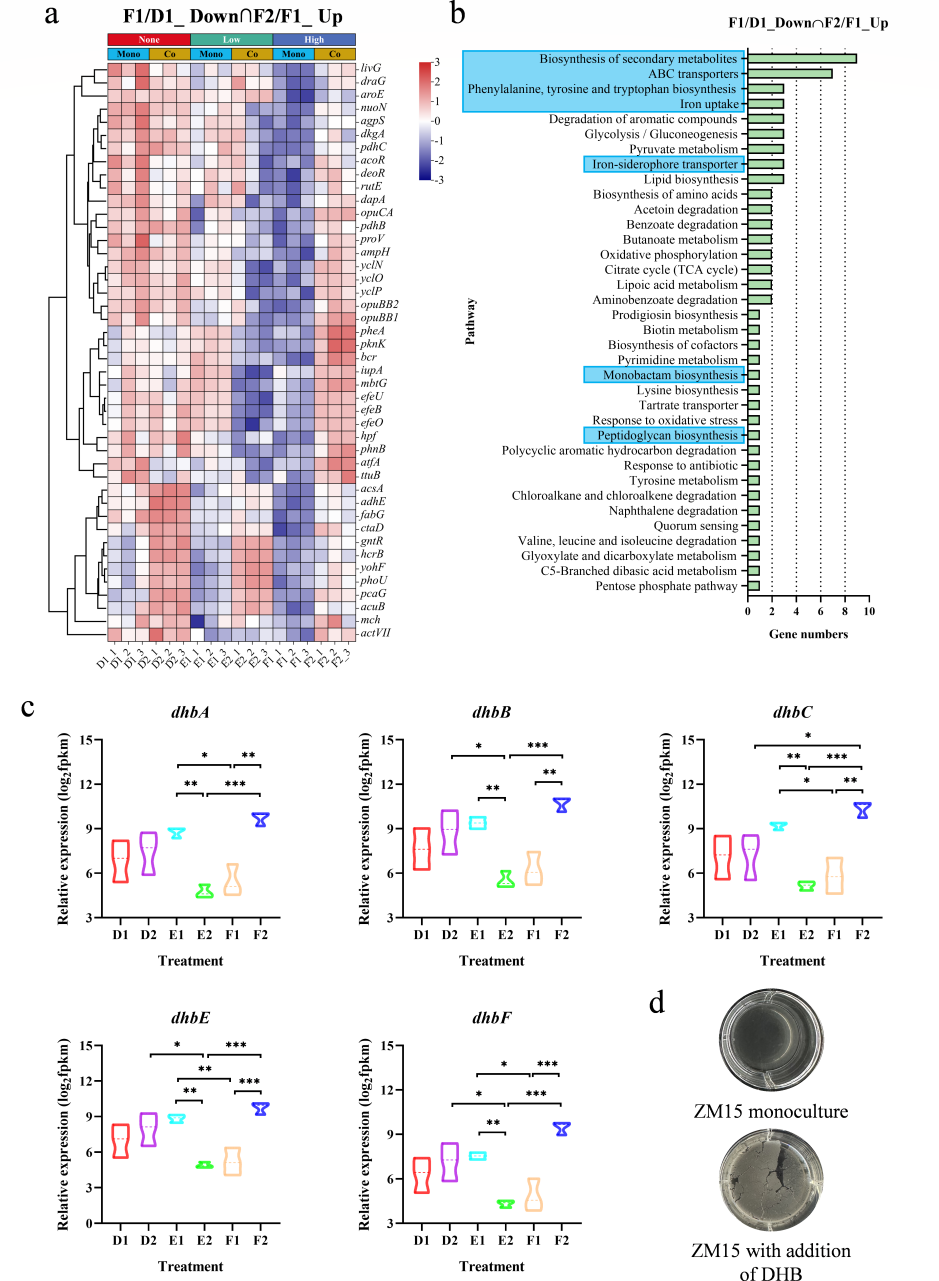
(a) Heatmap analysis of the genes downregulated under hyperosmotic stress (F1 vs D1) but upregulated in coculture (F2 vs F1). (b) KEGG pathway analysis of these DEGs that showed opposite trends under hyperosmotic stress and in coculture. (c) Relative expression of the *dhbABCEF* gene cluster. Significance tests were performed using a two-tailed *t*-test via GraphPad Prism 8.0.1. The symbol *** indicates *P* < 0.001, ** indicates *P* < 0.01, and * indicates *P* < 0.05. Each group had three biological replicates. (d) The growth of *R. ruber* ZM15 monoculture and with the addition of low concentration (0.2% of the DEHP molar concentration) of DHB.

Third, we found that *E. zeae* ZM18 stimulated the transport of iron ions and compatible solutes by *R. ruber* ZM15 in coculture, which promoted biofilm formation and significantly restored growth capacity. By analyzing the pathways in which these 71 DEGs (F2 vs F1) were involved, we found that hyperosmotic stress-repressed genes involved in the transport of iron ions as well as compatible solutes, whereas their expression levels were restored in coculture (Fig. 4a and b). In recent decades, many studies have reported that siderophore-mediated iron acquisition plays a relevant role in biofilm formation by increasing intracellular iron levels to activate biofilm matrix synthesis (40–42). In *R. ruber* ZM15, genes associated with iron transport (*efeBOU*, *iupABC*) (Fig. 4a) and siderophore biosynthesis (*dhbABCEF*) (Fig. 4c) were significantly upregulated in coculture. Through exogenous addition experiments of 2,3-dihydroxybenzoate (DHB), a precursor for siderophores, we found that siderophores could promote biofilm formation in *R. ruber* ZM15 under hyperosmotic stress (Fig. 4e). This suggested that in coculture, *E. zeae* ZM18 stimulated the iron-mediated regulation of biofilm formation in *R. ruber* ZM15. In conclusion, we found that bacteria may respond to hyperosmotic stress by promoting cofactor synthesis, whereas interspecies interactions may promote biofilm formation and significantly restore growth capacity through cofactor exchange and stimulation of iron ion transport.

### Synergistic coculture facilitated biofilm formation in *R. ruber* ZM15 under hyperosmotic stress by metabolic cross-feeding

To elucidate the potential interspecific metabolic exchanges between *R. ruber* ZM15 and *E. zeae* ZM18 under hyperosmotic stress, cell-free supernatants of monocultured and cocultured samples under hyperosmotic stress were collected to determine the extracellular metabolomic profiles by using an untargeted HPLC-MS/MS-based metabolomic approach. A total of 714 metabolites were identified in the monoculture and coculture metabolomes. The metabolites that were more abundant in coculture but were less abundant in monoculture were assumed to be secreted by *E. zeae* ZM18 and might be utilized by *R. ruber* ZM15. Metabolites with opposite changes in content were assumed to be metabolized by *E. zeae* ZM18. The results showed that the extracellular metabolic environments differed under different osmotic conditions (Fig. S11a). The extracellular content of 89 differentially abundant metabolites was elevated in mono- or coculture (Fig. S11b). These 89 differentially abundant metabolites could be classified into 3 categories, among which the metabolite content of cluster 1 was elevated in coculture (E2 vs E1, F2 vs F1). The metabolites of cluster 1 were mainly enriched in metabolites involved in amino acid metabolism and cofactor synthesis (Fig. S11c and d), suggesting that they were likely secreted by *E. zeae* ZM18 and shared with *R. ruber* ZM15. The metabolites of cluster 2 and cluster 3, which were considered secreted by *R. ruber* ZM15 in response to hyperosmotic stress (Fig. S11d), were mainly enriched in metabolites involved in starch and sucrose metabolism and riboflavin metabolism.

**Fig 5:**
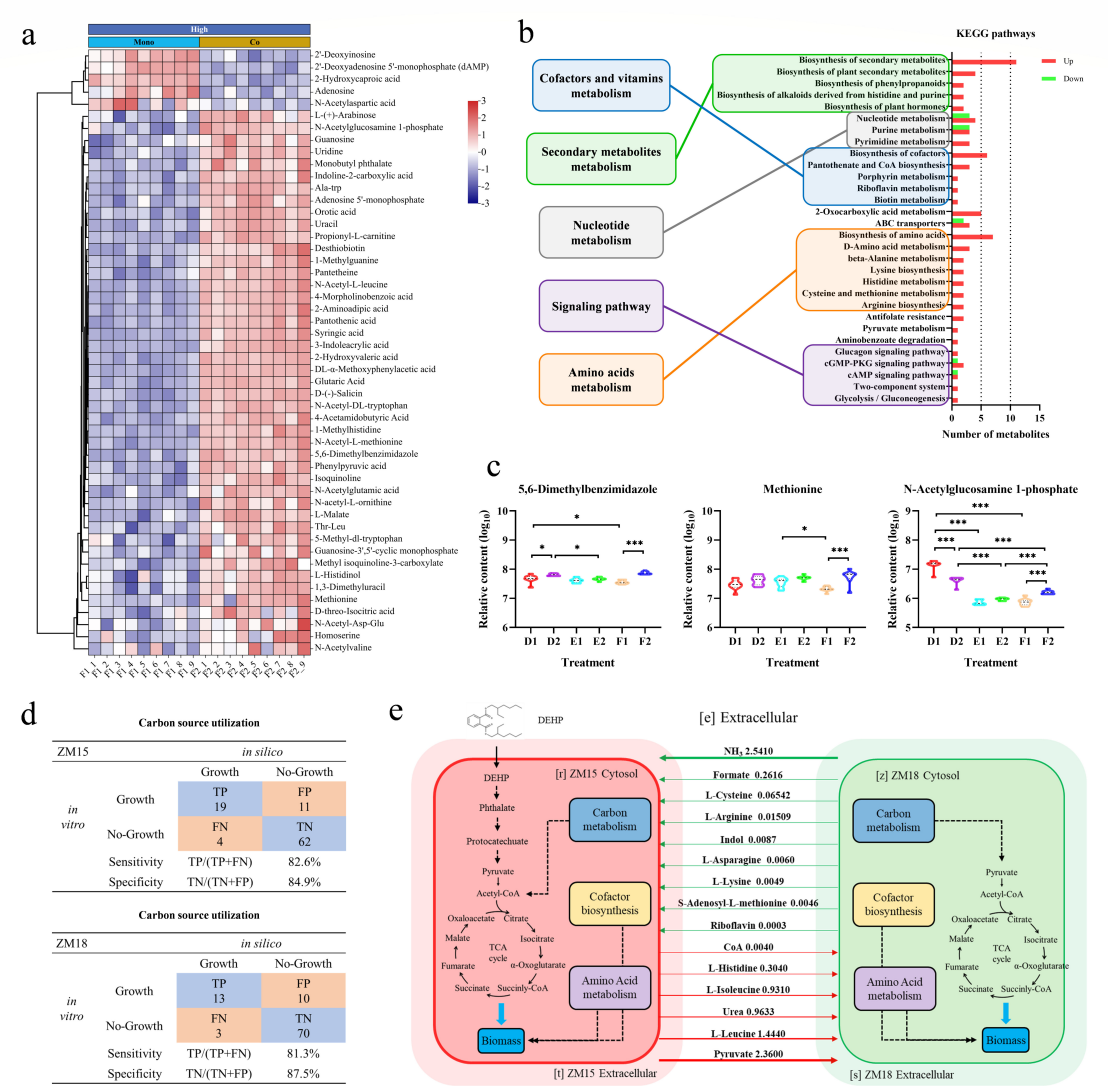
Metabolic cross-feeding in the synergistic consortium under hyperosmotic stress. (a) Metabolic profiles of the monoculture and coculture under hyperosmotic stress. The heatmaps show the log_10_ (relative abundance) values of each differentially abundant metabolite. The data were normalized using StandardScaler, and the Ward’s clustering method was used for heatmap analysis. (b) KEGG pathway analysis of the differentially abundant metabolites comparing coculture with monoculture under hyperosmotic stress. (c) Violin plots of the selected metabolites possibly related to cross-feeding. Significance tests were performed using a two-tailed *t*-test via GraphPad Prism 8.0.1. The symbol *** indicates *P* < 0.001, and * indicates *P* < 0.05. (d) Comparison of carbon source utilization of *R. ruber* ZM15 and *E. zeae* ZM18 *in vitro* and *in silico*. TP, true positive; FP, false positive; TN, true negative; FN, false negative. (e) *In silico* predicted exchanged metabolites of the synergistic consortium by the two-species GEM. The green/red arrow indicates that the substance is secreted by *E. zeae* ZM18/*R. ruber* ZM15 and was absorbed by *R. ruber* ZM15/*E. zeae* ZM18. The number above the arrow represents the interspecies flux, and the arrow thickness is proportional to the flux.

The top 50 differential compounds (|log_2_ Fold Change| > 1, *P* < 0.05, VIP > 1) were selected based on fold change (F2 vs F1) and relative abundance (Fig. 5a). The differentially abundant metabolites were mainly concentrated in cofactors and vitamins, secondary metabolites, nucleotides, and amino acids (Fig. 5a and b), consistent with the results of the transcriptomic analysis. As a result, most of these differentially abundant metabolites were upregulated in coculture under hyperosmotic stress, among which 5,6-dimethylbenzimidazole, methionine, and N-acetylglucosamine 1-phosphate were potentially secreted by *E. zeae* ZM18 and subsequently utilized by *R. ruber* ZM15 (Fig. 5a through c). Moreover, *R. ruber* ZM15 could produce nonanoic acid, N-acetyl-DL-phenylalanine, and quinolinic acid, which might be metabolized by *E. zeae* ZM18 for growth (Fig. S12). The exchange of 5,6-dimethylbenzimidazole, which is involved in the biosynthesis of vitamin B_12_, and methionine, which is involved in the methionine-folate cycle, suggested a specific contribution of the B_12_-dependent methionine-folate cycle to the hyperosmotic stress tolerance of the consortium, which is consistent with the transcriptomic analysis. N-acetylglucosamine 1-phosphate is one of the precursors for the biosynthesis of ECA, and the increased amount of N-acetylglucosamine 1-phosphate in coculture suggested that *E. zeae* ZM18 might promote biofilm formation in the consortium by sharing precursors for biofilm matrices with *R. ruber* ZM15. In addition, many nucleotides were found among the extracellular metabolites, suggesting that eDNA may also serve as an important component of the biofilm of this consortium (Fig. 5a; Fig. S12). Although transcriptome and metabolome analyses have revealed complex metabolic exchanges and interactions in the consortium, the large amount of data has blurred the focus, so we applied metabolic modeling to further elucidate the metabolic exchanges in the consortium from a global perspective to obtain definitive results.

The metabolic cross-feeding hypothesis was supported by a genome-scale metabolic model. The single-strain GEMs of *R. ruber* ZM15 and *E. zeae* ZM18 were reconstructed separately, and the accuracy of the final models was validated by the consistency between the phenotype arrays (Biolog PM1 plate) and *in silico* prediction of growth on alternate carbon sources (Fig. 5d; Fig. S13; Supplemental Data Set). A two-species metabolic model was reconstructed based on single-strain GEMs, and pFBA was used to predict the most likely exchanged metabolites in DEHP-MSM (Supplemental Data Set). The simulation results showed that *R. ruber* ZM15 and *E. zeae* ZM18 potentially shared 29 metabolites with each other (Fig. 5e; Table S5), most of which were detected in their original or derivatized forms by untargeted HPLC-MS/MS. Among the 29 predicted exchanged metabolites, 5 and 11 were cofactors and amino acids, respectively (Table S5). Although amino acid exchange fluxes were much greater than cofactor exchange fluxes (Fig. 5e), based on the omics data, we presumed that the cofactors SAM and RIBF, which are involved in vitamin B_12_ biosynthesis, were the key exchanged metabolites between species.

### Supplementation of key exchanged metabolites revealed physiological benefits of cross-feeding

**Fig 6:**
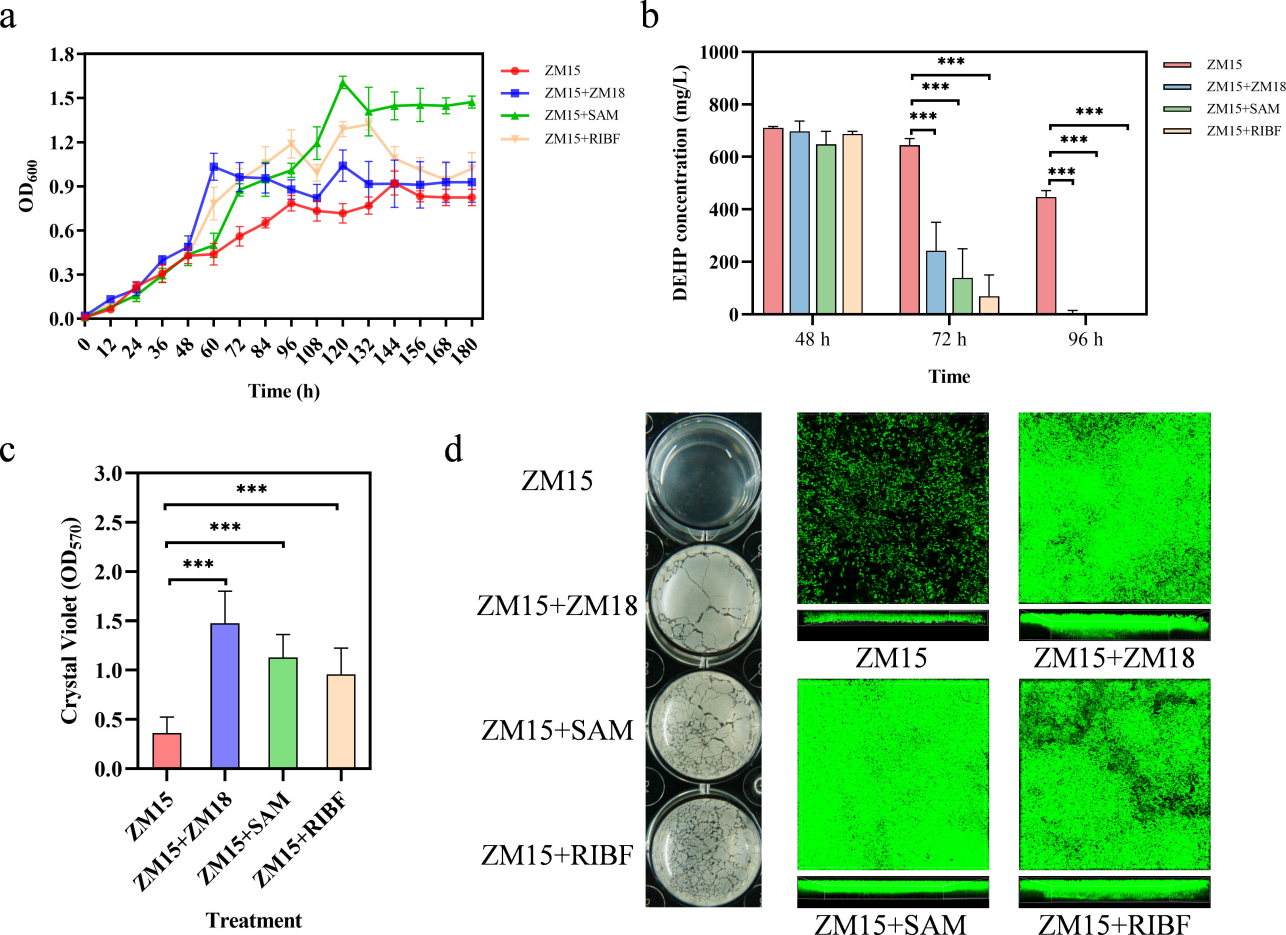
*In vitro* validation of the *in silico* predicted exchanged compounds in the synergistic consortium. (a) Comparison of the growth curve of *R. ruber* ZM15 in DEHP-MSM supplemented with predicted compounds under hyperosmotic stress (5% NaCl). (b) DEHP residual concentration of the different treatments. Each group had three biological replicates (**a, **b). Significance tests were performed using a two-tailed *t*-test via GraphPad Prism 8.0.1. The symbol *** indicates *P* < 0.001. (c) The amount of biofilm formation of *R. ruber* ZM15 in different treatments was determined by the crystal violet method. Significance tests were performed using a two-tailed *t*-test via GraphPad Prism 8.0.1. The symbol *** indicates *P* < 0.001. Each group had three biological replicates. (d) The biofilms of the different treatments were cultivated in microplates and visualized by CLSM.

Further validation assays showed that the addition of predicted key exchanged metabolites could enhance DEHP degradation by *R. ruber* ZM15 under hyperosmotic stress. To test whether SAM and RIBF could promote the growth and DEHP degradation of *R. ruber* ZM15, *R. ruber* ZM15 cells were cultivated in DEHP-exposed MSM supplemented with low concentrations (0.2% of the DEHP molar concentration) of SAM or RIBF under hyperosmotic stress, respectively. The higher biomass and lower residual DEHP content in monocultures with supplementation (Fig. 6a and b) validated the importance of these two compounds for *R. ruber* ZM15 growth and DEHP degradation. The *in vitro* results confirmed that *in silico* predicted key exchanged compounds could be utilized by *R. ruber* ZM15 and suggested that these two compounds might be responsible for the promotion of DEHP degradation by *E. zeae* ZM18 under hyperosmotic stress in the coculture system. Furthermore, the biofilms of *R. ruber* ZM15 supplemented with SAM and RIBF under hyperosmotic stress conditions were denser and thicker, as expected (Fig. 6c and d). This result suggested that SAM and RIBF promoted biofilm formation under hyperosmotic stress and alleviated hyperosmotic stress in *R. ruber* ZM15.

## DISCUSSION

In natural environments, microbes are usually exposed to various environmental stresses (43), such as hyperosmolarity, that can disrupt cellular homeostasis and interfere with essential cellular functions. The mechanisms of bacterial hyperosmotic stress tolerance at the single-cell level have been well studied (15). Many studies have demonstrated that synthetic microbial communities are more capable of promoting plant hyperosmotic stress tolerance than single strains (11, 44). However, it is still unclear how bacteria in communities collaborate with one another to tolerate hyperosmolarity, particularly the role of metabolic interactions in this context. Microbial degradation of pollutants is currently a hot topic, and as previously reported, ubiquitous hyperosmolarity in contaminated environments could inhibit bacterial growth and disrupt bioremediation processes (45, 46). Therefore, positive interactions among microbes during pollutant degradation might support their survival under hyperosmotic stress.

As reported in this study, we constructed a binary synergistic consortium composed of a highly efficient DEHP degrader, *R. ruber* ZM15, and a good cooperative partner, *E. zeae* ZM18, to assist *R. ruber* ZM15 in hyperosmotic stress tolerance. To comprehensively understand the hyperosmotic stress response and microbial interaction mechanism of this synergistic consortium, an integrated analysis of the transcriptome, metabolome, and metabolic model was performed. Through transcriptome and metabolome analysis, we hypothesized that vitamin B_12_ might be critical for bacterial hyperosmotic stress tolerance, and further metabolic modeling simulations revealed interspecies exchange of the cofactors SAM and RIBF in coculture, which are needed for vitamin B_12_ synthesis. *In vitro* experiments confirmed that both SAM and RIBF could significantly improve the tolerance of DEHP-degrading bacterium to hyperosmotic stress, demonstrating that common intracellular cofactors might play important roles in bacterial synergy and stress tolerance.

### Interspecies cross-feeding leads to the strong stress tolerance of this synergistic consortium

*Rhodococcus* isolates have a broad variety of accessible substrates and excellent environmental stress tolerance (47), making *Rhodococcus* an ideal type of bacteria for biotechnological applications, such as biodegradation and industrial production, given their wide range of metabolic processes (48, 49). However, the degradation efficiency of *R. ruber* ZM15 was greatly reduced under hyperosmotic stress in this study. Therefore, elucidating its mechanism of adaptation to hyperosmotic stress is important for improving the efficiency of biodegradation and may shed light on the mechanism of hyperosmotic stress tolerance in other microorganisms. Recently, GEMs have been reconstructed for three *Rhodococcus* strains, *R. jostii* RHA1 (50), *R. opacus* PD630 (51), and *Rhodococcus* sp. HS-D2 (52). The GEM for *R. ruber* ZM15 in this study was reconstructed based on genome annotation and the high-quality GEM for *R. jostii* RHA1. Ultimately, the phenotype prediction accuracy of this GEM exceeded 80% (Fig. 5d, Supplemental Data Set); therefore, we considered its predictions to be reasonably reliable. This is the first GEM for *R. ruber*, increasing the availability of GEMs for the genus *Rhodococcus*.

*Epilithonimonas zeae* was transferred from *Chryseobacterium zeae* (53), a species for which research is currently lacking. Therefore, it is currently unknown what ecological functions this species can perform in environmental remediation. In this study, we explored the role of this poorly known species in environmental remediation and the bacterial hyperosmotic stress response and constructed the first metabolic model of *E. zeae*, which serves as a useful reference for other researchers to construct metabolic models of similar species. We found by drop plate experiments that *E. zeae* ZM18 is not a salt-tolerant strain; instead, it is tolerant to acid stress (pH 5.5, Fig. S5). During the degradation of many organic pollutants, the production of intermediate metabolites such as protocatechuic acid may result in acidic cultures (29, 54). Therefore, acid tolerance may enable *E. zeae* ZM18 to survive better in this coculture system. Given the poor performance of *R. ruber* ZM15 and *E. zeae* ZM18 in monoculture under hyperosmotic conditions, we hypothesize that interspecies positive interactions are key to hyperosmotic stress tolerance in the consortium and that metabolic exchanges from *E. zeae* ZM18 play a decisive role in enhancing the hyperosmotic stress tolerance of *R. ruber* ZM15.

### Cofactors play an important role in bacterial stress tolerance

According to the above hypothesis that metabolic exchange is critical for hyperosmotic stress tolerance, despite the sophisticated interspecies interactions in the consortium, the combination of multi-omics, metabolic modeling, and exogenous addition assays confirmed that *R. ruber* ZM15 and *E. zeae* ZM18 collaborate through complex metabolic exchange (Fig. 3 to 6). As an indispensable methyl donor, SAM facilitates methylation reactions that recycle adenine and methionine and contributes to the production of the global quorum-sensing signal autoinducer-2. In addition, SAM is necessary for the synthesis of N-acetyl homoserine lactone, vitamins, and other biomolecules produced by SAM radical reactions (55). It has been reported that methionine limitations significantly impair the biofilm formation of *Staphylococcus aureus* and *Pseudomonas aeruginosa* (56). We therefore suggest that the B_12_-dependent methionine-folate cycle is important for biofilm formation and that it might affect biofilm formation through a variety of pathways, including via an increase in SAM for quorum sensing and an increase in dTTP for EPS synthesis. Previous studies have reported that the synthesis of the siderophore albomycin is dependent on the radical SAM (57, 58). We found that coculturing relieved the inhibition of iron transport under hyperosmotic stress, suggesting that the B_12_-dependent methionine-folate cycle may also be involved in iron-siderophore synthesis.

Although the predicted exchanges from transcriptomic and metabolomic analyses do not exactly match the predictions of the metabolic model, the major interaction mechanisms revealed were in good agreement. For example, some of the differentially regulated genes and metabolites in the transcriptome and metabolome were not reflected in the GEM predictions. On the one hand, this may be due to incomplete genome annotation resulting in missing metabolic reactions. On the other hand, the transcriptome can only reflect a moment of bacterial physiology and biochemistry and cannot reflect the continuous cumulative process of bacterial biochemistry. In addition, pFBA has limitations, in that it is only applicable to the simulation of steady-state fluxes. pFBA does not consider kinetic parameters or the role of gene expression regulation. Furthermore, model simulations did not consider the metabolic burden imposed by biofilm formation on cellular metabolism. The additional benefits of biofilm to microorganisms also cannot be modeled. Therefore, the predictions may not always be accurate. This indicated that the predictions obtained by each method alone had a degree of specificity, and a comprehensive application of multiple methods is conducive to obtaining more accurate predictions. The key mechanisms are revealed layer by layer and confirmed from different perspectives with different methods, through which we can piece together a more complete picture based on the fragmented results obtained simultaneously.

### Vitamin B_12_ might affect biofilm formation in *R. ruber* ZM15

Moreover, we wondered whether the positive effects of vitamin B_12_ on biofilm formation and bacterial hyperosmotic stress tolerance were case specific. The salt-induced compatible solute betaine was recently found to promote the production of vitamin B_12_ (59), but how vitamin B_12_ affects bacterial hyperosmotic stress tolerance remains elusive. Vitamin B_12_ has been reported to be essential for biofilm formation by *P. aeruginosa* (60), and its effects may vary depending on the situation at hand. Vitamin B_12_ was able to increase biofilm development when class II ribonucleotide reductase (RNR) activity was suppressed. However, exogenous addition of vitamin B_12_ decreased biofilm formation during anaerobic respiration (61). In *R. ruber* ZM15, GM004055 (*nrdJ*), which encodes a vitamin B_12_-dependent RNR, was involved in DNA biosynthesis, and its expression decreased with increasing salt concentration but increased in coculture (data not shown). We therefore suggest that the effect of vitamin B_12_ on biofilms may not be coincidental and that vitamin B_12_ likely exerts an important effect on biofilm formation through a complex metabolic pathway.

In addition, many studies have indicated the importance of vitamin B_12_ in microbial stress tolerance. For example, vitamin B_12_ could enhance the thermal tolerance of *Chlamydomonas reinhardtii* by increasing B_12_-dependent methionine synthesis (62). The vitamin B_12_ mutant strain of *Listeria monocytogenes* was more sensitive to low temperature and copper stress than the wild-type strain (63). Recent research has shown that high-temperature stress might select for species capable of promoting mutualism through vitamin B_12_ sharing (6). Zhao et al. demonstrated that silver nanoparticles prevented the evolution of drug resistance in *P. aeruginosa* by accelerating B_12_-dependent folate and methionine cycles, which re-established DNA synthesis and partially counteracted high oxidative stress levels (64). Based on the above reported results and our research findings, it is reasonable to assume that vitamin B_12_ contributes significantly to microbial tolerance. Regrettably, we were unable to knock out the synthetic genes of these key cofactors in *R. ruber* ZM15 and *E. zeae* ZM18 to further demonstrate the relationship between vitamin B_12_ and hyperosmotic stress tolerance, although we tried many times with different methods. We believe that this may be due to the relatively low recombination efficiency of *R. ruber* ZM15 and *E. zeae* ZM18, resulting in too few transformants to screen for the correct mutants. Type strains could be used in further investigations to validate the role of the B_12_-dependent methionine-folate cycle in bacterial hyperosmotic stress tolerance. The present study may draw attention to the role of cofactors and the B_12_-dependent methionine-folate cycle in hyperosmotic stress tolerance mechanisms. We believe that further understanding the hyperosmotic stress response and microbial interaction mechanisms can provide promising strategies for environmental remediation, which will be the direction of our future research.

### Conclusions

In conclusion, our findings demonstrated that cofactor exchange played a key role in the complex synergy of the DEHP-degrading consortium, complementing the role of cofactors in microbial tolerance and environmental remediation strategies. Although the current understanding of the role of vitamin B_12_ in hyperosmotic stress tolerance is still in its infancy, a previously unknown mechanism for metabolic interactions between bacterial species is highlighted here. More importantly, an exploration of either vitamin B_12_-eliciting microbial synergy or exogenous vitamin B_12_ application could offer promising alternatives to microbial hyperosmotic stress tolerance.

## SUPPLEMENTAL MATERIAL

Supplemental information msystems.00884-24-s0001.docx Supplemental tables and figures.

Supplemental data sets msystems.00884-24-s0002.xlsx Data Sets S1 to S5.

Supplemental legends msystems.00884-24-s0003.docx Legends for supplemental data sets and supplemental information.

ASM does not own the copyrights to Supplemental Material that may be linked to, or accessed through, an article. The authors have granted ASM a non-exclusive, world-wide license to publish the Supplemental Material files. Please contact the corresponding author directly for reuse.
