# Supplementary material for: Interspecies synergistic interactions mediated by cofactor exchange enhance stress tolerance by inducing biofilm formation
Source: mSystems. 2024 Aug 27;9(9):e00884-24. doi: 10.1128/msystems.00884-24 (PMC11406921; doi:10.1128/msystems.00884-24)
Supplement: Supplemental information — Supplemental tables and figures. [file msystems.00884-24-s0001.docx]

Supplementary information

**Interspecies synergistic interactions mediated by cofactor exchange enhance stress tolerance by inducing biofilm formation**

**Authors:**

Lvjing Wang^1,2^, Xiaoyu Wang^1,2^, Hao Wu^1,2^, Haixia Wang^3^, Zhenmei Lu^1,2*^

1. MOE Laboratory of Biosystem Homeostasis and Protection, College of Life Sciences, Zhejiang University, Hangzhou 310058, China

2. Cancer Center, Zhejiang University, Hangzhou 310058, China

3. College of Biotechnology and Bioengineering, Zhejiang University of Technology, Hangzhou 310058, China

* Address correspondence to Zhenmei Lu, [lzhenmei@zju.edu.cn](mailto:lzhenmei@zju.edu.cn)

**Supplementary file contents**

**Supplementary tables1**

Table S11

Table S22

Table S33

Table S44

Table S56

**Supplementary figures7**

Figure S17

Figure S28

Figure S310

Figure S411

Figure S512

Figure S613

Figure S714

Figure S815

Figure S916

Figure S1017

Figure S1118

Figure S1219

Figure S1320

# Supplementary tables

Table S1. Sequences of primers for qRT-PCR used in this study.

| Primer | Sequence (5’ > 3’) |
| --- | --- |
| qPCR-*bluB*-F/qPCR-*bluB*-R | CATCAAGGTCGAAGGCATC/GAGCCACAGGTTCTGGAT |
| qPCR-*cobT*-F/qPCR-*cobT*-R | GACCTGGGAATGCGTCTC/GAACGTCGCCATGTTCGT |
| qPCR-*cobV*-F/qPCR-*cobV*-R | GATCGGGGTGGGATTTCT/CCGACTTCATCACCTCAC |
| qPCR-*cobP*-F/qPCR-*cobP*-R | GGATTCCGAGATCGAGATG/GTACTCCCCGACTGTCAG |
| qPCR-*cobN*-F/qPCR-*cobN*-R | GTTCTCGTTCAAGGAATT/GAGTGTTTCGTCGGATAC |
| qPCR-*cobG*-F/qPCR-*cobG*-R | GTTTCCTCTTCGGGCTCG/GATCGTGACGCGGAACAG |
| qPCR-*cobH*-F/qPCR-*cobH*-R | ACGAGGTGCTGTGCTTCC/GTGGGTGCGTTGCCGATC |
| qPCR-*cobJ*-F/qPCR-*cobJ*-R | GTGCTGGGCATCTACAAC/GAGGATCAGGCACTTCAT |
| qPCR-*cobM*-F/qPCR-*cobM*-R | CTGGACCTGGACGAGATC/GAATACAGCGACGGGTCA |
| qPCR-*cobL*-F/qPCR-*cobL*-R | CTGATCGTGCTCTCCTCC/GATGCAGACGACGTTGAG |
| qPCR-*cobO*-F/qPCR-*cobO*-R | GGTGTTCCAATTCGTCAAG/CATCTTGTGCCACTCGAT |
| qPCR-*cobB*-F/qPCR-*cobB*-R | CTGTACGACGGGATGCTC/GCCTTGTTCAGGATCACC |
| qPCR-*cobD*-F/qPCR-*cobD*-R | ACGACGTGCTCAATCTGG/GACGACACCGTGATAGAC |
| qPCR-*cobK*-F/qPCR-*cobK*-R | ATGACGGTGCTCGTTCTC/GGTACTGTTGCAGTCCCT |
| qPCR-*cobQ*-F/ qPCR-*cobQ*-R | GGCGGATTCCAGATGCTC/CGTGGTGGATCTCGTAAC |

Table S2. The top 11 genes with the highest correlation with phenotype in the green module.

| Gene | Correlation |
| --- | --- |
| GM003106 | 0.988841878 |
| GM004084 | 0.983842387 |
| GM005286 | 0.982501532 |
| GM002473 | 0.98081395 |
| GM003519 | 0.980300771 |
| GM003039 | 0.975116517 |
| GM002248 | 0.974916926 |
| GM004078 | 0.97446849 |
| GM000627 | 0.972993619 |
| GM002575 | 0.964231984 |
| GM004090 | 0.964199594 |

Table S3. Co-up/downregulated genes of *R. ruber* ZM15 under both low and hyperosmotic stress.

| Gene id | Subject description | Pathway | Up or down |
| --- | --- | --- | --- |
| GM000360 | Band 7 domain-containing protein |  | Down |
| GM000776 | Serine protease | Lipid metabolism | Down |
| GM001180 | Ectoine hydroxylase, *ectD* | 5-Hydroxyectoine biosynthesis | Up |
| GM002143 | D-3-phosphoglycerate dehydrogenase, *serA* | L-serine biosynthesis | Up |
| GM002454 | Conserved exported hypothetical protein | Transporters | Down |
| GM002814 | Hypothetical protein |  | Down |
| GM003482 | Lactoylglutathione lyase, *gloA* | Methylglyoxal degradation | Up |
| GM003543 | Conserved exported hypothetical protein | Transporters | Up |
| GM003721 | Transcriptional regulatory protein DevR (DosR), *devR* | Two-component system | Down |
| GM003722 | Sensor histidine kinase | Two-component system | Down |
| GM003872 | Resuscitation-promoting factor RpfC, *rpfC* | Glycan metabolism | Up |
| GM004084 | ChlD component of cobalt chelatase involved in B12 biosynthesis, *chlD* | Vitamin B12 biosynthesis | Up |
| GM004138 | PE-PGRS family protein | Stress response; Virulence | Down |
| GM004139 | Hsp70 family protein | Stress response | Down |
| GM004148 | Alkane 1-monooxygenase 1, *alkB1* | Fatty acid degradation; Caprolactam degradation | Up |
| GM004566 | Magnesium and cobalt transport protein CorA, *corA* | Transporters | Up |
| GM005144 | UPF0053 protein, magnesium and cobalt exporter, *yfjD* | Transporters | Down |
| GM005285 | Putative acyl-[acyl-carrier-protein] desaturase DesA1, *desA1* | Fatty acid metabolism | Up |
| GM005286 | Universal stress protein, *usp* | Stress response | Up |
| GM005289 | Thymidylate synthase, *thyA* | Pyrimidine metabolism; dTTP biosynthesis | Up |

Table S4. Genes involved in the vitamin B_12_ biosynthesis pathway.

| Gene id | Subject description |
| --- | --- |
| GM001061 | Vitamin B12 transport periplasmic protein BtuE |
| GM001062 | 5, 6-Dimethylbenzimidazole synthase |
| GM001356 | Cobalamin biosynthesis protein CobW |
| GM001979 | Cob(I)yrinic acid a, c-diamide adenosyltransferase |
| GM002235 | Uroporphyrinogen-III C-methyltransferase CysG |
| GM002293 | Cobalt ABC transporter ATP-binding protein |
| GM002696 | Precorrin-3B synthase |
| GM003061 | Nicotinate-nucleotide-dimethylbenzimidazole phosphoribosyltransferase |
| GM003062 | Adenosylcobinamide-GDP ribazoletransferase |
| GM003156 | Cobalamin biosynthesis protein |
| GM003261 | Cobalamin biosynthesis protein |
| GM003381 | Cobalamin biosynthesis protein CbiX |
| GM003382 | Bifunctional uroporphyrinogen-III synthetase/response regulator domain protein |
| GM003846 | Bifunctional uroporphyrinogen-III C-methyltransferase/uroporphyrinogen-III synthase |
| GM004043 | Adenosylcobinamide kinase/adenosylcobinamide phosphate guanylyltransferase |
| GM004045 | Cobalamin import system permease protein BtuC |
| GM004077 | Precorrin-6A synthase (deacetylating) |
| GM004078 | Aerobic cobaltochelatase subunit CobN |
| GM004079 | Cobalamin biosynthesis protein CobG |
| GM004080 | Precorrin-8X methylmutase |
| GM004081 | Precorrin-3B C (17)-methyltransferase |
| GM004082 | Precorrin-4 C (11)-methyltransferase |
| GM004083 | Precorrin-6Y C5, 15-methyltransferase |
| GM004085 | Cob(I)alamin adenolsyltransferase/cobinamide ATP-dependent adenolsyltransferase |
| GM004086 | Cobyrinic acid a, c-diamide synthase |
| GM004087 | Cobalamin biosynthesis protein |
| GM004089 | Cobalt-precorrin-6A reductase |
| GM004090 | Cobyric acid synthase CobQ |
| GM004101 | Cobalamin biosynthesis protein CobW |
| GM004357 | Putative Cobalamin synthesis CobW domain protein |

Table S5. List of metabolites potentially shared between *R. ruber* ZM15 and *E. zeae* ZM18 simulated by pFBA.

|  | From ZM18 to ZM15 | From ZM15 to ZM18 |
| --- | --- | --- |
| Amino acids | L-Lysine; L-Asparagine; L-Arginine; L-Cysteine; L-Glutamate | L-Histidine; L-Isoleucine; L-Leucine; L-Tyrosine; L-Glutamine; L-Proline |
| Carbohydrates | D-Fructose |  |
| Cofactors | Riboflavin; S-Adenosyl-L-methionine; Menaquinone | CoA; Menaquinone 7 |
| Organic acids | Formate | Pyruvate; Succinate |
| Nucleosides | Adenosine | Uridine |
| Others | Indole; NH_3_; Ornithine; Acetaldehyde; CO_2_ | Urea; H_2_O_2_ |

# Supplementary figures


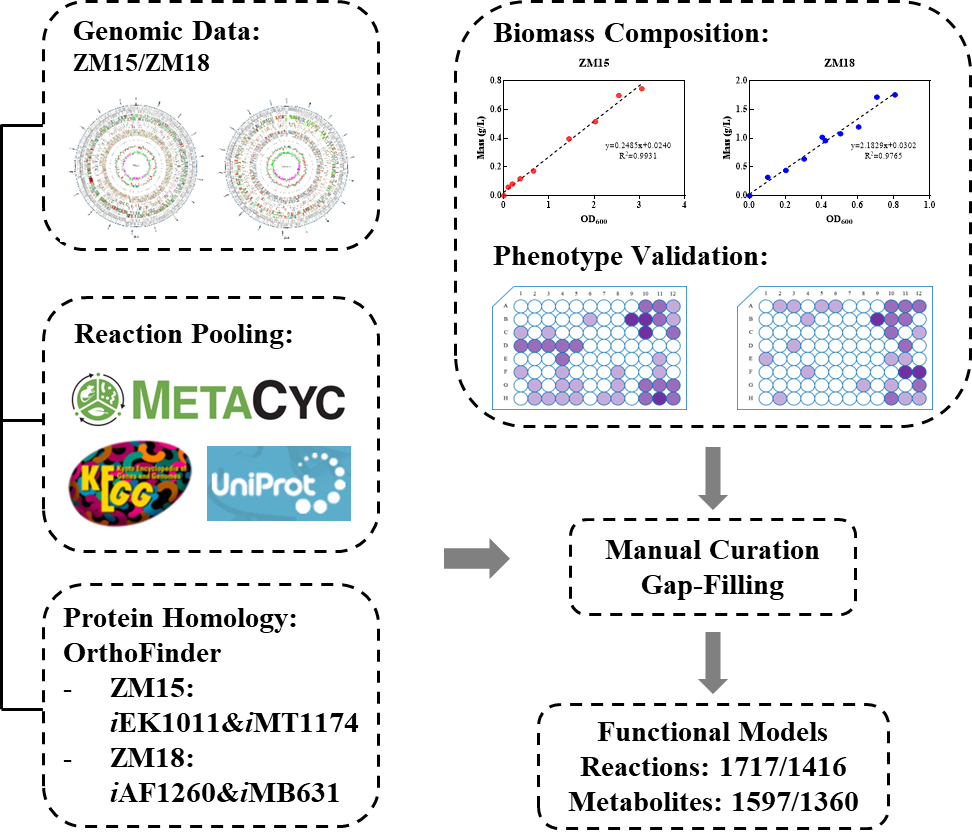


Figure S1. The details of the reconstruction and refinement process of GEMs in this study. Model quality was assessed by MEMOTE (https://github.com/opencobra/memote), but poor annotations of metabolites, reactions, and genes resulted in a low assessment score. Therefore, the accuracy of model predictions was verified by using the consistency of carbon source utilization phenotypes and model predictions.


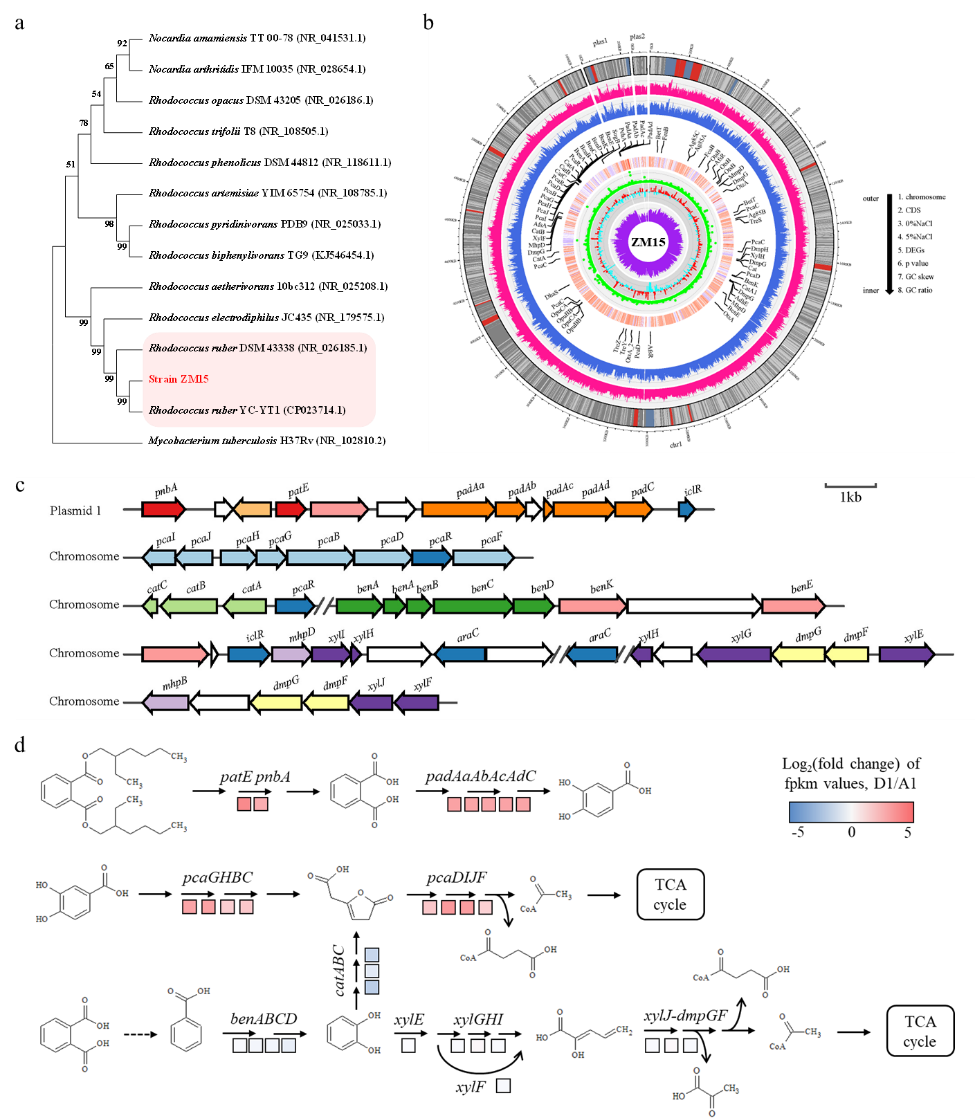


Figure S2. Phylogenetic analysis and global view of the *R. ruber* ZM15 genome and transcriptome. a. Phylogenetic analysis based on the 16S rRNA gene sequence. b. Global view of the *R. ruber* ZM15 genome and transcriptome under no salt and high salt conditions. Concentric circles from periphery to the core represent the following: (i) chromosomal location; (ii) genome component of *R. ruber* ZM15; the dark gray areas represent the positive strand coding genes; the light gray areas represent the negative strand coding genes; the red areas represent genomic islands and the blue areas represent prophages; (iii) bar charts in rose represent gene expression levels under no salt condition in DEHP-MSM; (iv) bar charts in blue represent gene expression levels under high salt condition in DEHP-MSM; (v) differentially expressed gene analysis, where the color indicates whether a gene is upregulated (red), downregulated (blue), or shows no significant change (white) under high salt condition, and the location of core genes in the genome is also indicated; (vi) scatter diagram showing the *p* values of DEGs; (vii) bidirectional bar charts showing the GC skew of the genome; (viii) GC ratio of the genome. c. DEHP degradation gene cluster in the *R. ruber* ZM15 genome. d. The proposed pathway for DEHP degradation by *R. ruber* ZM15 as inferred from the transcriptome. The boxes below the genes represent the corresponding genes annotated in the genome and their relative expression. A1: *R. ruber* ZM15 monoculture under no salt condition in LB (NaCl-free). D1: *R. ruber* ZM15 monoculture under no salt condition in DEHP-MSM.


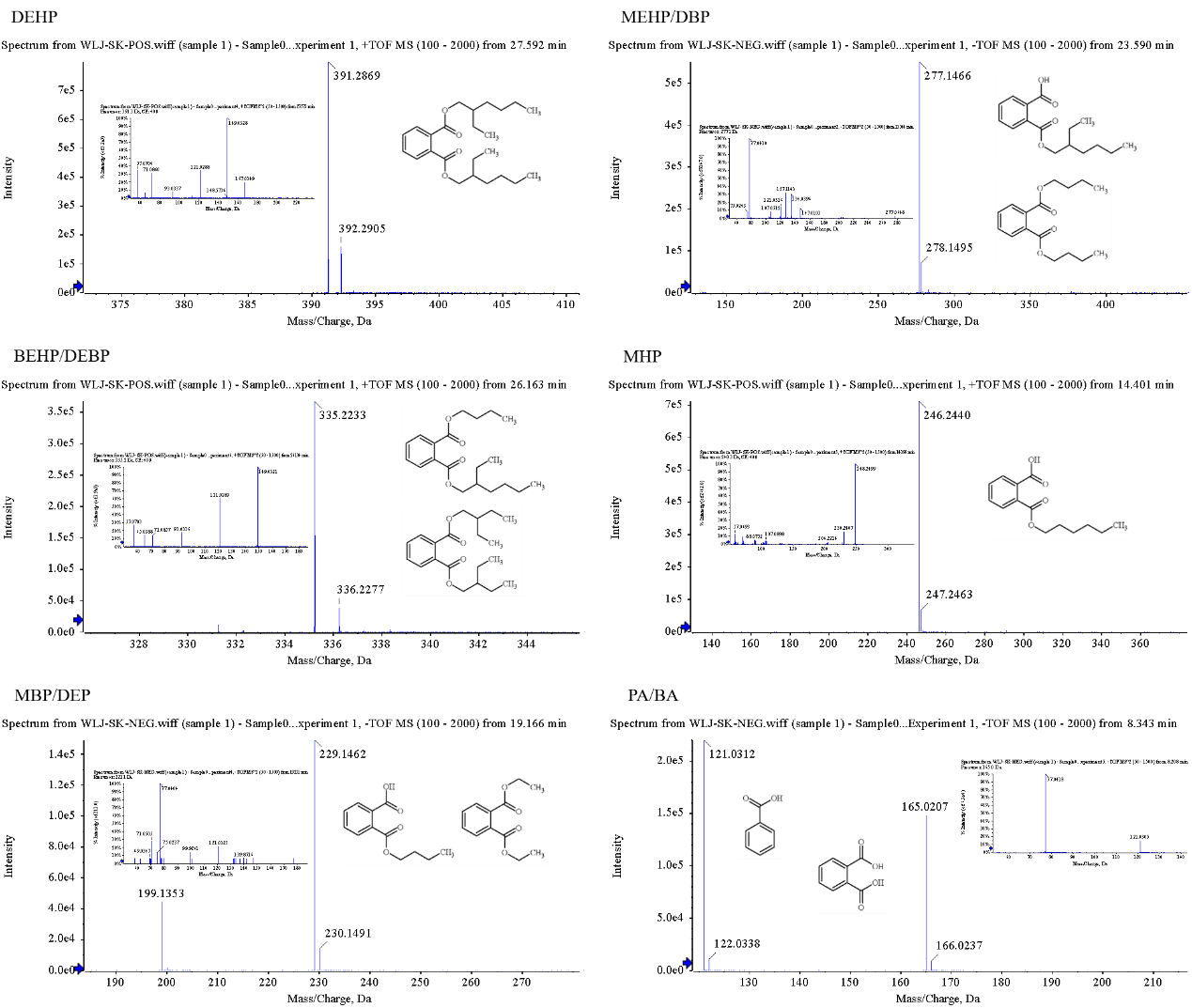


Figure S4. Detection of metabolic intermediates by LC-MS. Each large figure is a primary spectrum of the compound and the small figure is a secondary spectrum.


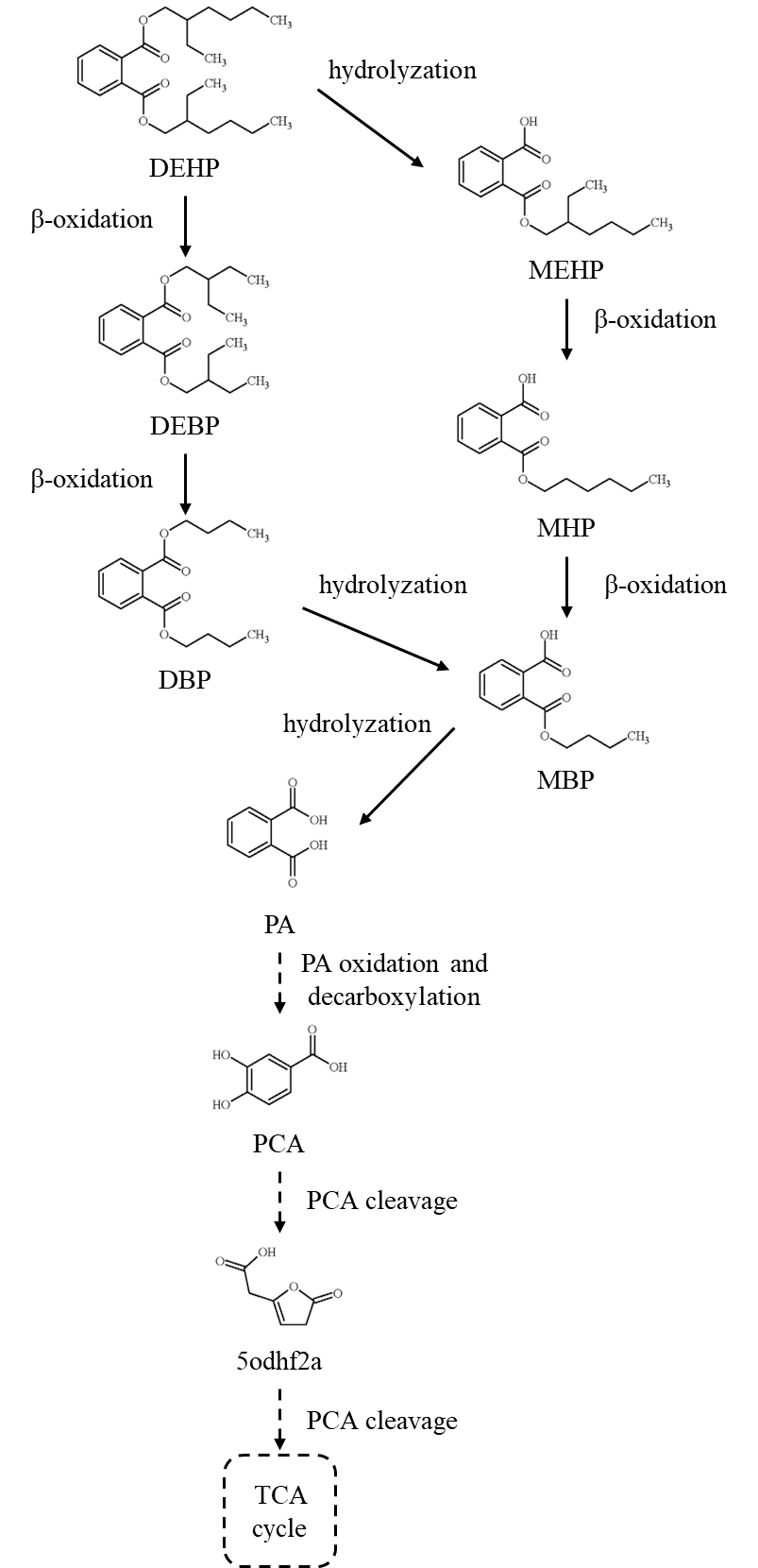


Figure S3. Proposed metabolic pathway of DEHP in *R. ruber* ZM15.

Figure S5. The environmental stress tolerance of *R. ruber* ZM15 and *E. zeae* ZM18 was tested by drop plate experiments. All stress tests were performed in LB medium except for the oligotrophic stress test (0.2% glucose). *R. ruber* ZM15 had excellent environmental stress tolerance in enriched medium and was able to tolerate a wide range of environmental stresses, whereas *E. zeae* ZM18 was less tolerant.


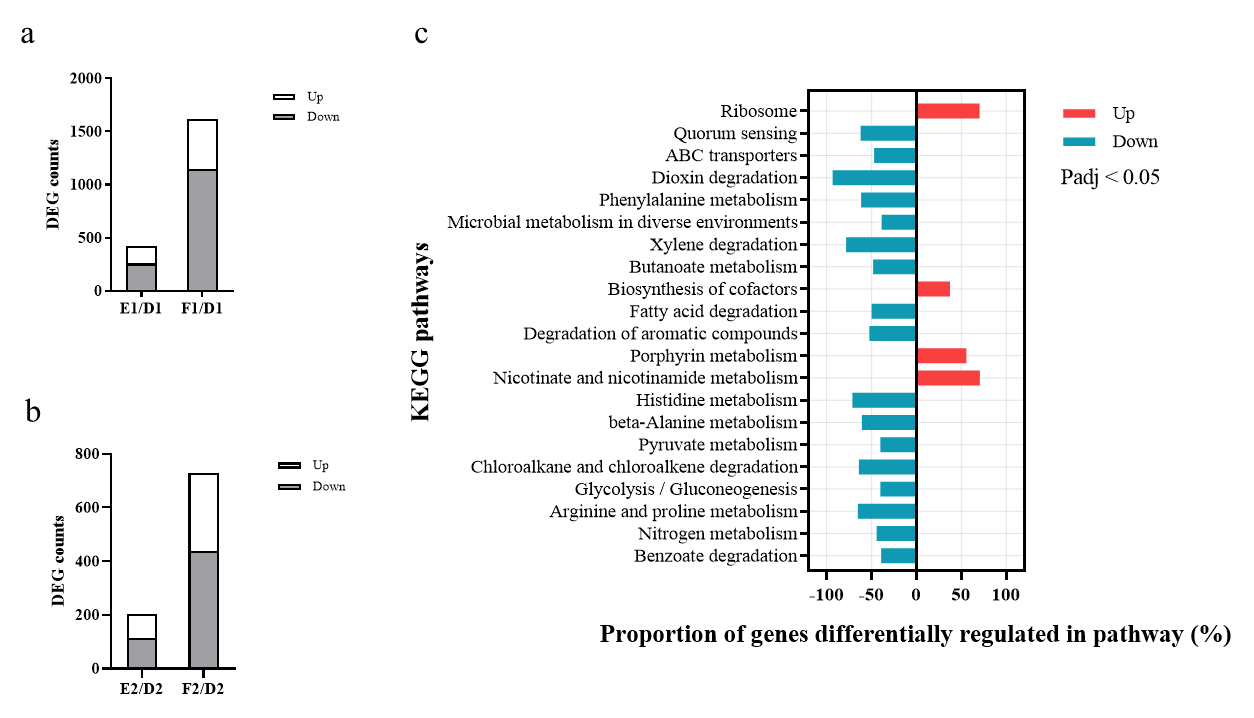


Figure S6. The number of DEGs of *R. ruber* ZM15 under low (2.5% vs. 0%) and high (5% vs. 0%) salt condition in monoculture (a) and coculture (b). D1: *R. ruber* ZM15 monoculture under no salt condition in DEHP-MSM; E1: *R. ruber* ZM15 monoculture under low salt condition (2.5% NaCl) in DEHP-MSM; F1: *R. ruber* ZM15 monoculture under high salt condition (5% NaCl) in DEHP-MSM; D2: *R. ruber* ZM15 coculture with *E. zeae* ZM18 under no salt condition in DEHP-MSM; E2: *R. ruber* ZM15 coculture with *E. zeae* ZM18 under low salt condition (2.5% NaCl) in DEHP-MSM; F2: *R. ruber* ZM15 coculture with *E. zeae* ZM18 under high salt condition (5% NaCl) in DEHP-MSM. (c) KEGG pathway analysis of the differentially expressed genes of *R. ruber* ZM15 induced (red) and regressed (blue) in response to hyperosmotic stress. The pathways shown from top to bottom are arranged in order from smallest to largest adjusted *p* value (Padj).


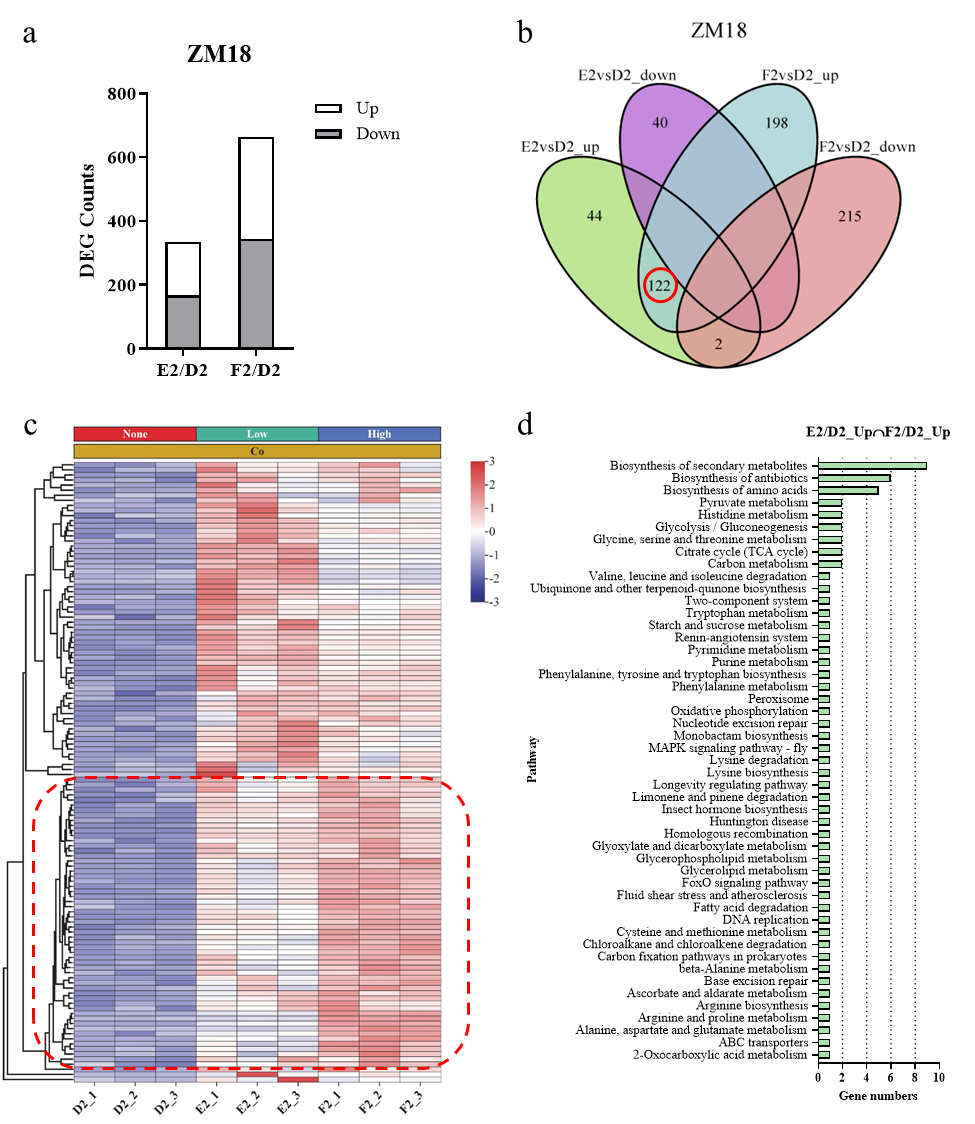


Figure S7. Transcriptional response of *E. zeae* ZM18 to hyperosmotic stress in the synergistic consortium. (a) The number of DEGs of *E. zeae* ZM18 under low (2.5% vs. 0%) and high (5% vs. 0%) salt condition in coculture. (b) In coculture, 122 genes of *E. zeae* ZM18 were up-regulated in both low and high salt treatments. (c) Heatmap analysis of the 122 co-upregulated genes under both low and high salt conditions in coculture. The heatmaps showed the log_2_ (fpkm) values of each DEG. The data were normalized using StandardScaler and the ward’s clustering method was performed in heatmap analysis. None indicates 0% NaCl salt condition, Low indicates 2.5% NaCl salt condition, and High indicates 5% NaCl salt condition. Co indicates *R. ruber* ZM15 and *E. zeae* ZM18 coculture group. (d) KEGG pathways involved in genes whose expression increased with salt gradient.


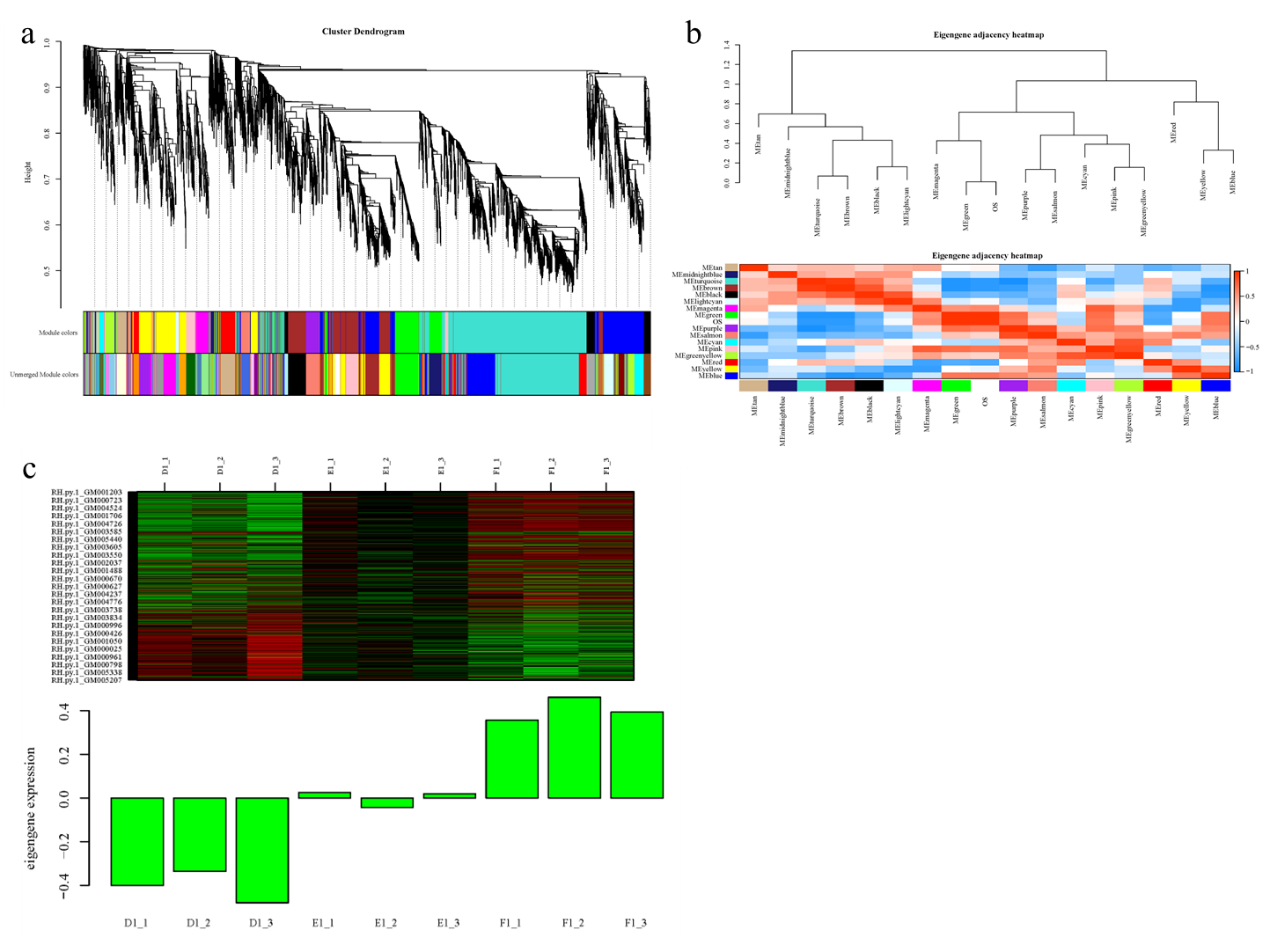


Figure S8. WGCNA of the *R. ruber* ZM15 transcriptome under hyperosmotic stress. (a) Cluster dendrogram of the *R. ruber* ZM15 transcriptome under hyperosmotic stress. In the dendrogram, each leaf represents a gene, and each module below the dendrogram is marked with a color. WGCNA identified 16 gene clusters of co-expressed genes under hyperosmotic stress. (b) Eigengene adjacency heatmap of the *R. ruber* ZM15 transcriptome under hyperosmotic stress. The heatmap represents the correlation between modules and phenotype OS (OS represents osmolality). Red represents a positive correlation, and blue represents a negative correlation. The green module has the strongest positive correlation with the phenotype. (c) Gene expression in the green module. Gene expression in this module varies with the salt gradient.


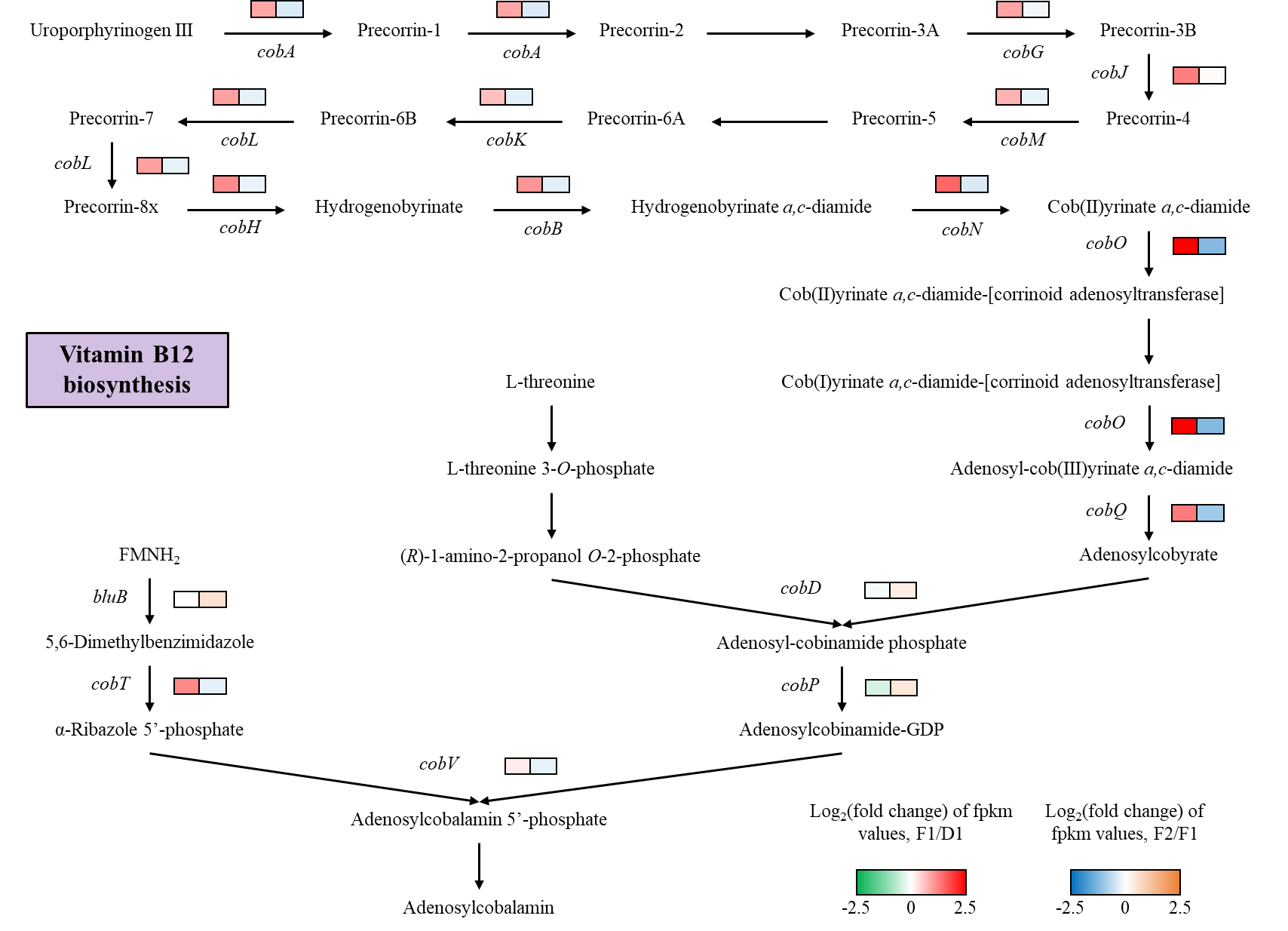


Figure S9. Changes in transcriptomic data of genes involved in the vitamin B_12_ biosynthetic pathway in *R. ruber* ZM15.


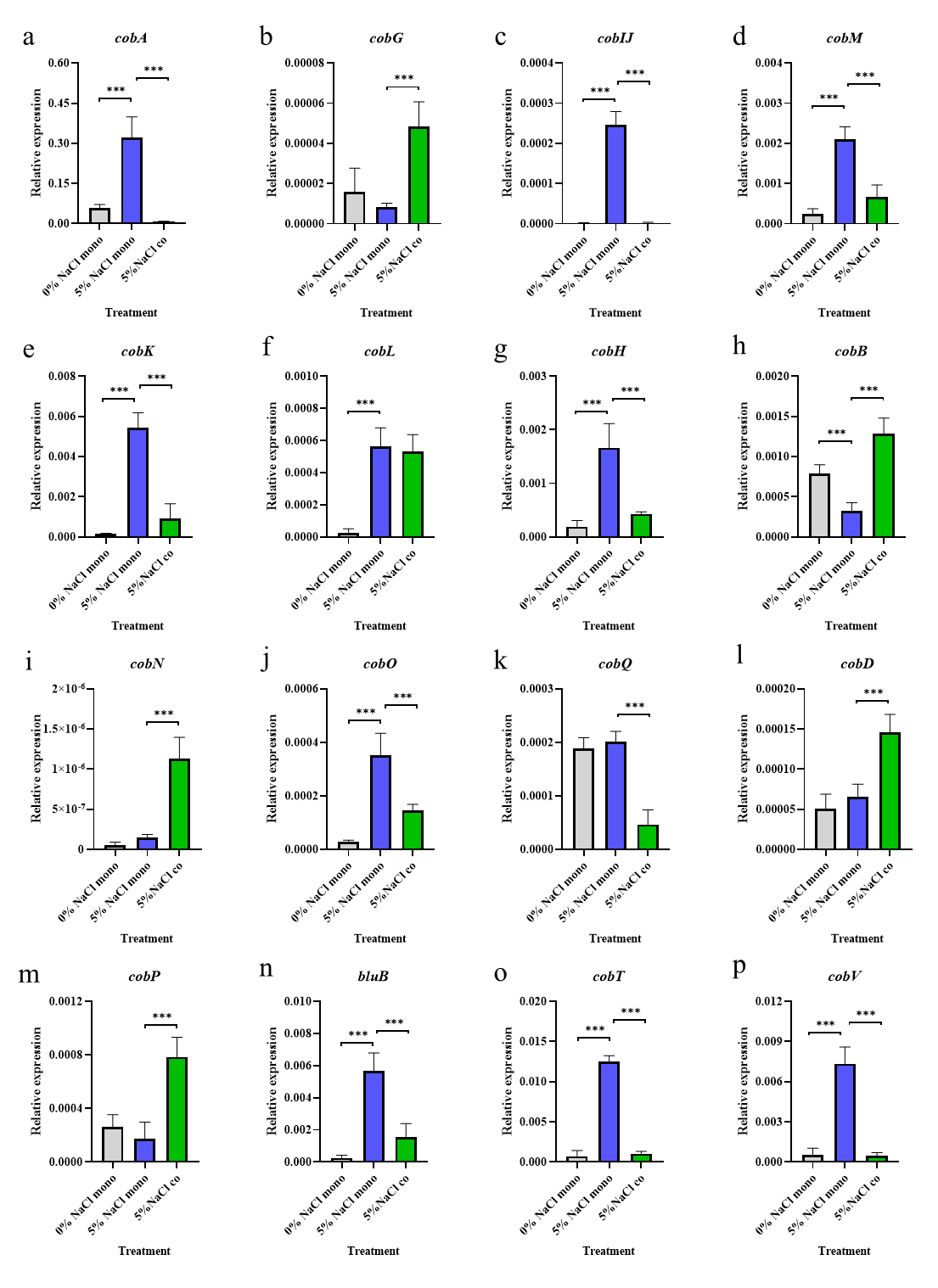


Figure S10. Relative expression of the genes involved in the vitamin B12 biosynthetic pathway in *R. ruber* ZM15. qRT-PCR analysis of the selected genes under different treatments (a-p). The expression of genes was normalized by 16S rRNA expression levels. Significance tests were performed using a two-tailed t-test via GraphPad Prism 8.0.1. The symbol *** indicates *p* < 0.001. Each group had four biological replicates.


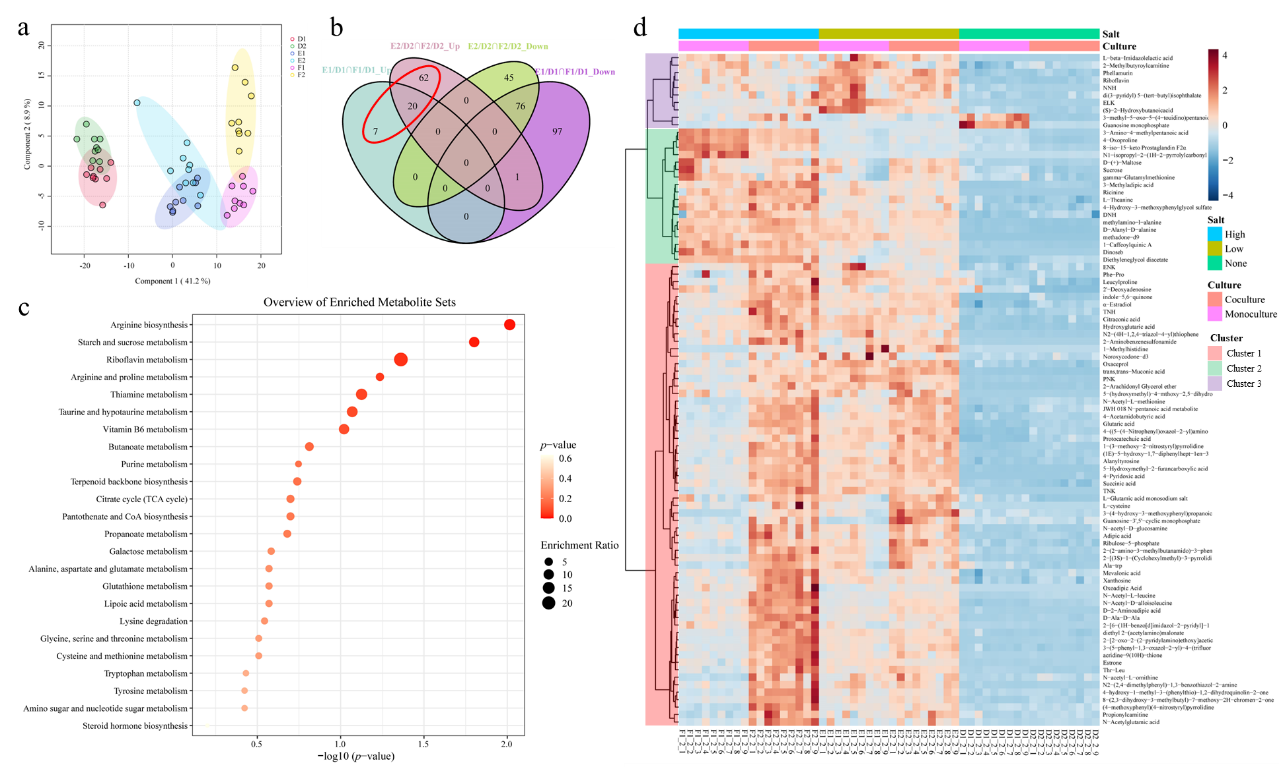


Figure S11. Extracellular metabolome analysis under different treatments. (a) Partial least squares discrimination analysis (PLS-DA) of metabolite composition was assessed by untargeted metabolomics in supernatants collected at different treatments using MetaboAnalyst 6.0. (b) Venn diagram depicting the overlap and distribution of differential metabolites (|log_2_ Fold Change| > 1, *p* < 0.05, VIP > 1). (c) Enriched KEGG metabolic pathway of 89 differential metabolites under osmotic stress using MetaboAnalyst 6.0. (d) Hierarchical clustering based on Euclidean distance of relative metabolite abundance of 89 differential metabolites. The heatmaps showed the log_10_ (relative abundance) values of each differential metabolite. The data were normalized using StandardScaler and the ward’s clustering method was performed in heatmap analysis.


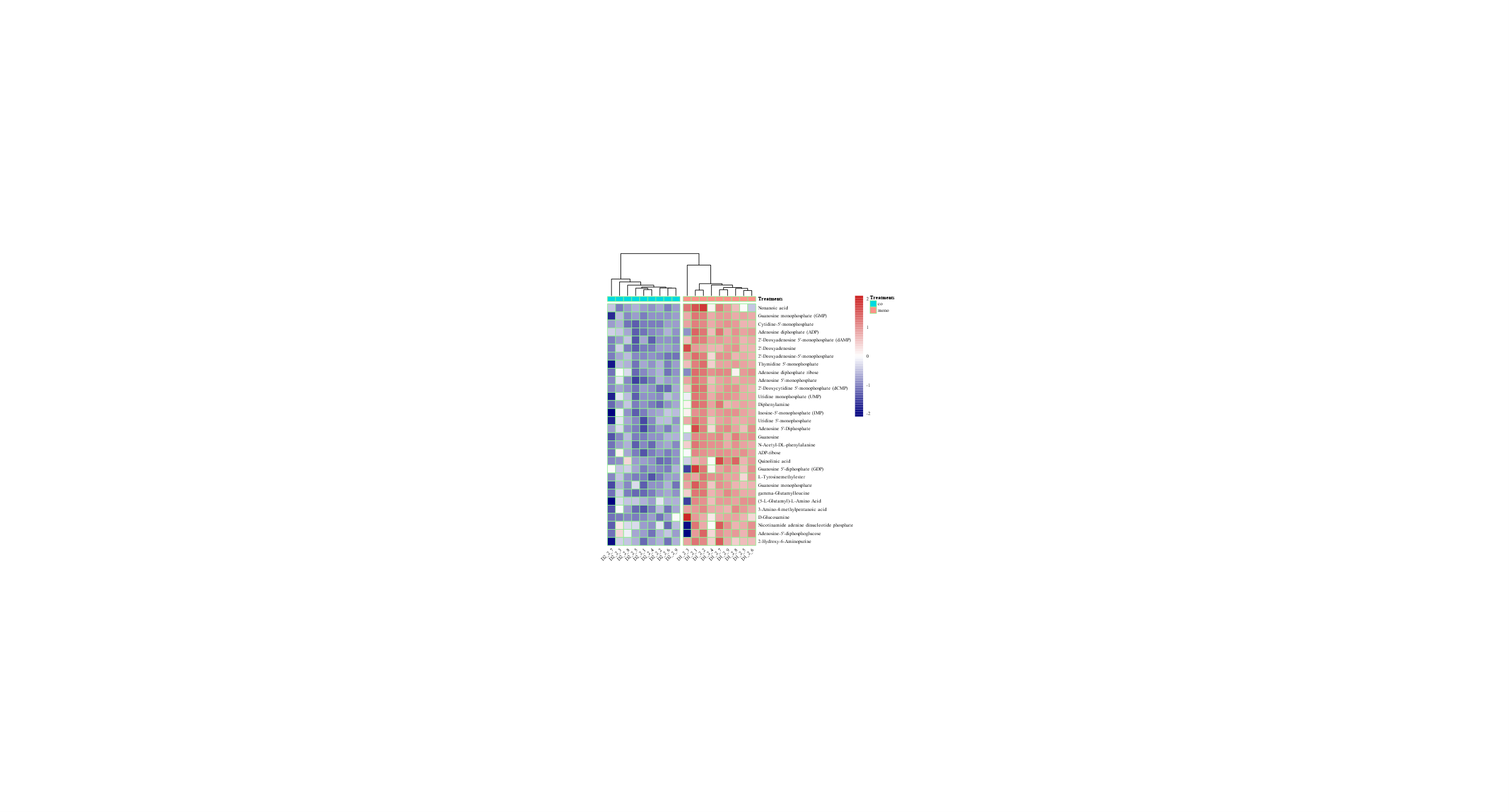


Figure S12. Heatmaps of the downregulated genes under coculture with no salt addition.


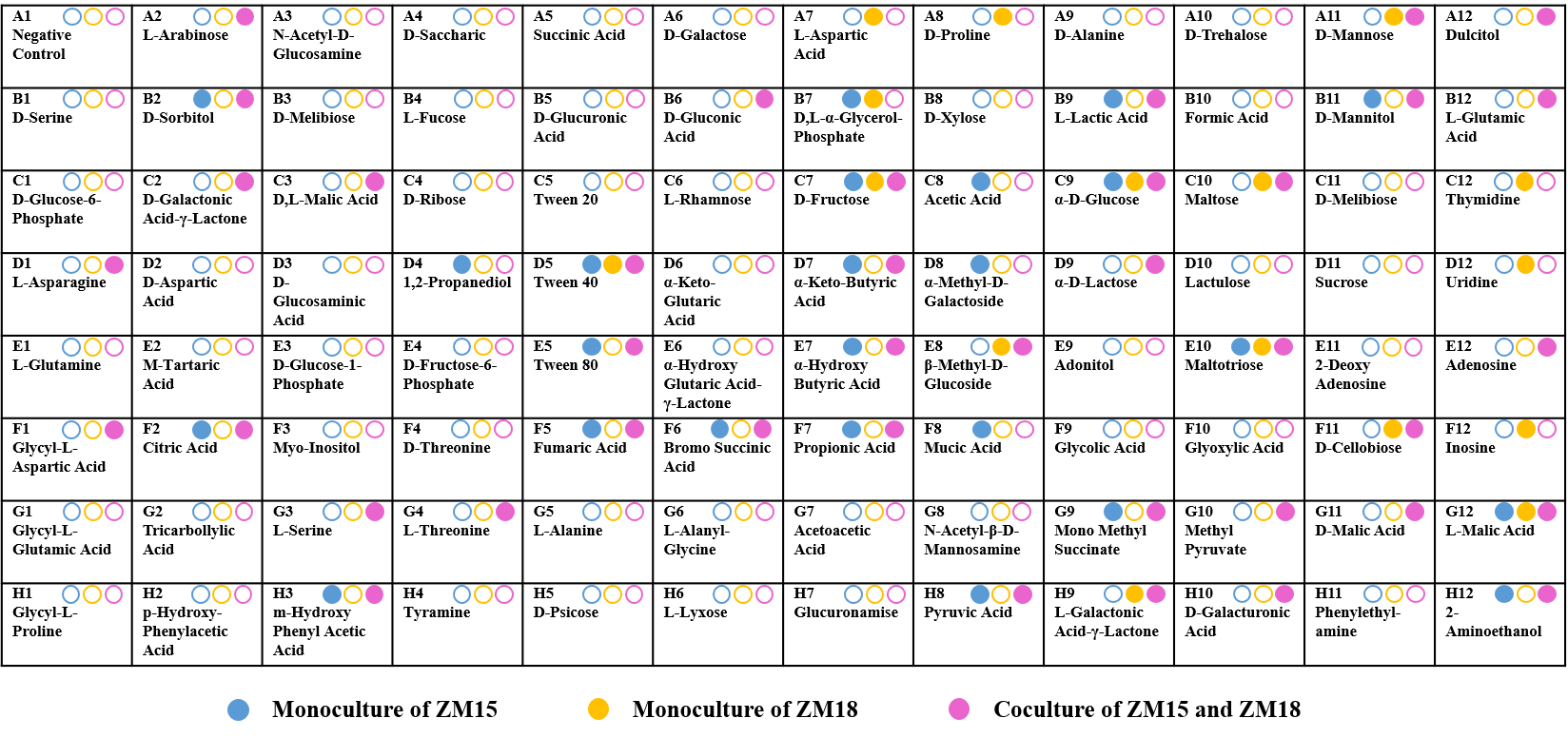


Figure S13. Biolog PM1 plates results for the two strains individually and in the consortium.
