# Supplementary material for: Interspecies synergistic interactions mediated by cofactor exchange enhance stress tolerance by inducing biofilm formation
Source: mSystems. 2024 Aug 27;9(9):e00884-24. doi: 10.1128/msystems.00884-24 (PMC11406921; doi:10.1128/msystems.00884-24)
Supplement: Supplemental legends — Legends for supplemental data sets and supplemental information. [file msystems.00884-24-s0003.docx]

**Supplemental legends**

**Legends for Supplemental dataset**

Supplemental dataset (xlsx format) contains two-species GEM data, where each sheet contains the data shown below.

Sheet 1: Dataset S1: two-species model (Reactions)

Sheet 2: Dataset S2: two-species model (Metabolites)

Sheet 3: Dataset S3: two-species model (pFBA)

Sheet 4: Dataset S4: exchange fluxes

Sheet 5: Dataset S5: carbon source test

**Legends for Supplementary information**

Supplementary information (docx format) contains supplementary tables (Tables S1-S5) and supplementary figures (Figures S1-S13).
